# The Arabidopsis holobiont: a (re)source of insights to understand the amazing world of plant–microbe interactions

**DOI:** 10.1186/s40793-023-00466-0

**Published:** 2023-02-17

**Authors:** M. J. Poupin, T. Ledger, R. Roselló-Móra, B. González

**Affiliations:** 1 Laboratorio de Bioingeniería, Facultad de Ingeniería y Ciencias, Universidad Adolfo Ibáñez, 7941169 Santiago, Chile; 2 Center of Applied Ecology and Sustainability (CAPES), Santiago, Chile; 3 Millennium Nucleus for the Development of Super Adaptable Plants (MN-SAP), Santiago, Chile; 4 Marine Microbiology Group, Department of Animal and Microbial Biodiversity, Mediterranean Institute for Advanced Studies (IMEDEA UIB-CSIC), Illes Balears, Majorca, Spain

**Keywords:** Arabidopsis, Bacteria, Community, Fungi, Microbiota, Plant, Plant-growth-promotion-rhizobacteria, Plant-root-exudates, Rhizosphere

## Abstract

As holobiont, a plant is intrinsically connected to its microbiomes. However, some characteristics of these microbiomes, such as their taxonomic composition, biological and evolutionary role, and especially the drivers that shape them, are not entirely elucidated. Reports on the microbiota of *Arabidopsis thaliana* first appeared more than ten years ago. However, there is still a lack of a comprehensive understanding of the vast amount of information that has been generated using this holobiont. The main goal of this review was to perform an in-depth, exhaustive, and systematic analysis of the literature regarding the Arabidopsis–microbiome interaction. A core microbiota was identified as composed of a few bacterial and non-bacterial taxa. The soil (and, to a lesser degree, air) were detected as primary microorganism sources. From the plant perspective, the species, ecotype, circadian cycle, developmental stage, environmental responses, and the exudation of metabolites were crucial factors shaping the plant–microbe interaction. From the microbial perspective, the microbe-microbe interactions, the type of microorganisms belonging to the microbiota (i.e., beneficial or detrimental), and the microbial metabolic responses were also key drivers. The underlying mechanisms are just beginning to be unveiled, but relevant future research needs were identified. Thus, this review provides valuable information and novel analyses that will shed light to deepen our understanding of this plant holobiont and its interaction with the environment.

**Supplementary Information:**

The online version contains supplementary material available at 10.1186/s40793-023-00466-0.

## Introduction

**Fig. 1:**
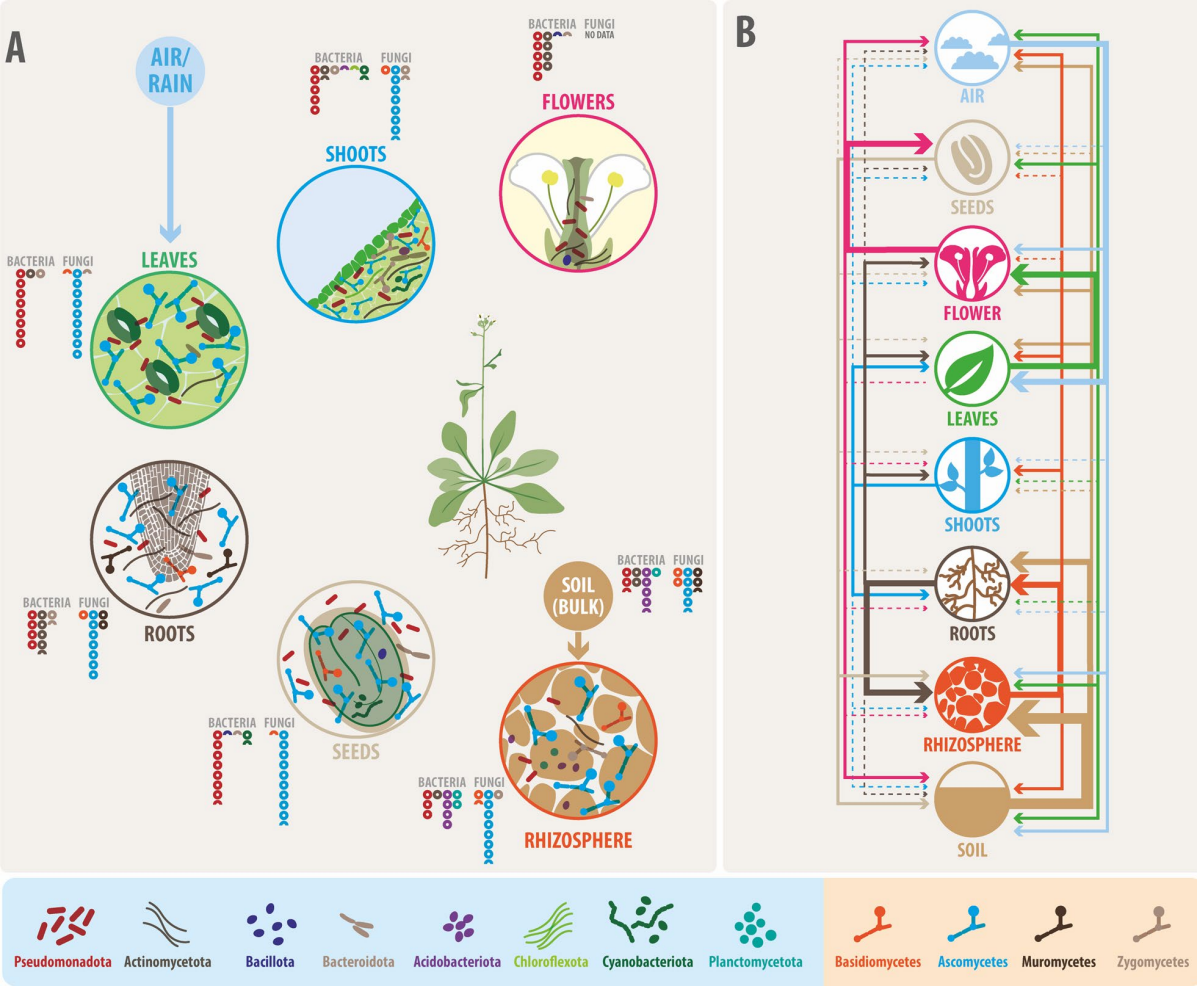
Features and connections in the Arabidopsis microbiota. **A** The distribution of the main microbial taxa among different plant compartments is represented by symbols explained in the boxes at the bottom of the figure; light blue for bacterial phyla and light orange for fungal phyla, while relative abundances of the major phyla are represented next to each compartment [20, 22, 23, 30, 60, 85, 191]. **B** Connections among the microbiota of compartments and their different sources (inputs) of inoculation. The arrow width represents the relative contributions of the sources based on the percentage of each source input with respect to the total input. Dashed lines represent minor influences. Roots comprise endorhizosphere plus rhizoplane. Leaves comprise the endophyllosphere plus the epiphyllosphere. Names of the taxa are *Pseudomonadota*, *Actinomycetota*, *Bacillota*, *Bacteroidota*, *Acidobacteriota*, *Chloroflexota*, *Cyanobacteriota*, *Planctomycetota*, *Basidiomycota*, *Ascomycota*, *Zygomycota*, and *Mucoromycota*, formerly *Proteobacteria*, *Actinobacteria*, *Firmicutes*, *Bacteroidetes*, *Acidobacteria*, *Chloroflexi*, *Cyanobacteria*, *Planctomycetes*, *Basidiomycetes*, *Ascomycetes*, *Zygomycetes,* and *Mucoromycetes*, respectively

The realization that all animal and plant species harbor complex associated microbial communities (the microbiota) in their surfaces as well as and inner parts is relatively recent in Biology [17]. Furthermore, the holistic view of the holobiont represented by the conjunction of a macro-organism and its microbiome (the associated microorganisms and their collective genomes) is even newer [26]. For decades, our understanding of these inter-kingdom interactions increased thanks to studies of plant or animal models interacting with single microbial species, such as pathogens and, to a lesser degree, beneficial microorganisms. However, the characteristics of the microbiome in experimental model species regarding their taxonomic composition, biological role, and especially the drivers that shape those microbiomes are far from being completely understood. Plants are holobiont harboring microorganisms in their internal and external tissues [43, 65, 72, 192] (Fig. 1A). Therefore, plant fitness, environmental responses, adaptation, and evolution should be addressed, considering plants as complex dynamic entities controlled by the hologenome: the host genome plus all the genomes of the microbiome.

**Fig. 2:**
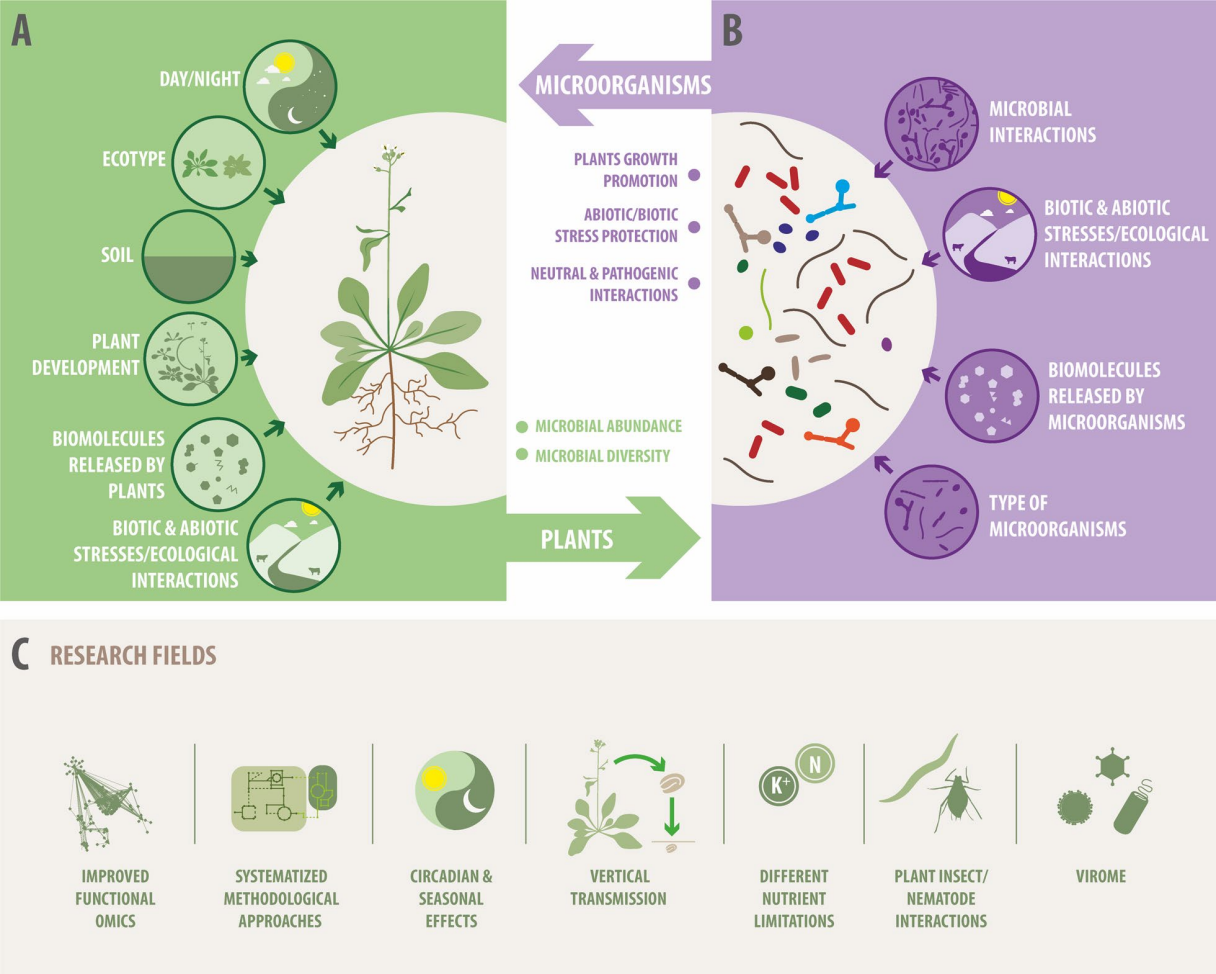
The Arabidopsis-microbiota interaction as a complex system: Multiple drivers that shape the outcomes of this plant–microbe interaction. **A** Drivers from the macro-organism plant perspective (from top to bottom): circadian and seasonal effects; ecotype; soil; plant developmental stage; biomolecules released by plants (i.e., root exudates); responses to the environment (i.e., biotic/abiotic stress responses) **B** Drivers from the microbial perspective: microbe-microbe interactions; microbial responses to biotic and abiotic stresses; biomolecules released by microorganisms and type of microorganisms (i.e., plant growth-promoting/protecting bacteria). **C** Future research needs

After rice (*Oryza sativa), Arabidopsis thaliana* is the second most studied plant species and has proven to be a valuable model in plant sciences [37, 144, 185], specifically to get insights into plant development and responses to the environment [92, 144]. As with other well-studied biological models, a few studies targeting *A. thaliana* interactions with bacterial populations were available at the beginning of this century, mainly using culture-dependent molecular approaches (e.g., [63, 91]. Since then, a great deal of information has been obtained with culture-independent molecular techniques and, more recently, with omics approaches. Massive sequencing allows statistically significant comparisons among plant compartments using Operational Taxonomic Units (OTUs) or a more accurate manually supervised clustering of Operational Phylogenetic Units [135]. More than 170 original reports have covered several aspects of this holobiont, such as the composition and structure of the microbial communities; comparisons between different compartments of the plant; abiotic and biotic factors controlling the shape of these communities, and, more recently, they have started the incipient elucidation of the underlying molecular mechanisms (Fig. 1A-B; Fig. 2A-B). Nevertheless, there is still a lack of a comprehensive understanding of the large amount of information generated using this holobiont (Fig. 2C). In this article, a thorough and systematic revision of the existing literature was performed, extracting common patterns, identifying some methodological or operative aspects, and proposing future research needs. Some of the aspects that are discussed are the composition of the core microbiome and its variation according to changes in the environment or plant compartments and traits; how the endophyte core community in Arabidopsis has similarities with those found in other plant species (confirming the valuable role of this species as a model for plant holobiont); the sometimes-controversial issue about the presence of microorganisms in plant seeds; and the multiple layers affecting microbe-microbe and plant-microbiota interactions (Fig. 2). This comprehensive and systematic analysis of the bulk of information already available in the well-studied Arabidopsis model will highlight critical aspects of plant-microbiome interactions, providing valuable guidelines for studying other plant hosts and their microbial communities.

Among these outcomes, the concept of core microbiota has been stablished for Arabidopsis, showing that it harbors community members that are essentially constant [30, 115, 169], while others are variably affected by the soil type, ecotype, developmental stage [115], season [3, 15, 30], generation [183], and other factors playing minor contributions (Fig. 2A). The root endophyte core community in Arabidopsis has the same most abundant phyla reported for many plant endophyte communities [70], confirming the valuable role of this species as a model for plant holobiont.

In addition, several reports provide clear evidence that the soil (both abiotic and biotic components) is the main factor explaining the root microbiota composition in Arabidopsis, and other plants [30, 47, 65, 115, 169, 183, 184, 194].

Nevertheless, there still needs to be a comprehensive understanding of the large amount of information generated using this holobiont (Fig. 2C). For example, the microbiome-root-shoot axis has been conceptualized and addressed (e.g., [80, 179]. The ways in which these compartments are connected are not sufficiently studied. One way is the external connection between microbes associated with the rhizosphere that are transferred (by proximity, early development contact, wind moving, insects, among others) to the surface of the leaves (Fig. 1B). The reverse way, always external, is through raindrops falling off from leaves to the rhizosphere and then to the root compartment (Fig. 1B). An alternative mechanism is through inner connections between the endorhizospheric- and endophyllospheric compartments.

In this article, a thorough and systematic revision of the existing literature was performed, extracting common patterns, identifying some methodological or operative aspects, and proposing future research needs. This comprehensive and systematic analysis of the bulk of information already available in the well-studied Arabidopsis model will highlight critical aspects of plant-microbiome interactions, providing valuable guidelines for studying other plant hosts and their microbial communities.

## Literature review methodology

PubMed and Google Scholar were browsed for eligible published articles up to December 2022, using the keywords: Arabidopsis, *A. thaliana*, microbiota, and microbiome. A few BioRxiv preprint files were included, as also milestone reviews on *A. thaliana* holobiont [31, 59, 62, 72, 136, 166]. Overall, nearly 220 research articles were analyzed to prepare the review and construct Additional file 1: Table SI. For taxonomy, the recently approved rules on phyla valid names are used [141]. Metabolic, functional, and ecological features linked to the plant biology of families or genera mentioned in the text are indicated in Table 1.

**Table 1:** Main metabolic, functional, and ecological features reported for microbial families and genera found associated with the Arabidopsis holobiont

Phylum	Family/Genus	Metabolic, functional, and/or ecological features	References^*^
*Bacteria*			
*Actinomycetota*	*Streptomycetaceae/Streptomyces*	Plant growth promotion. Biocontrol activity. Phosphate turnover. Antimicrobial synthesis	Olanrewaju and Babaloba (2019)
	*Nocardioidaceae/Nocardiodes*	Plant derived and pollutant compounds degradation. Plant growth promotion. Halophyte	Yang et al. (2020); Okazaki et al. (2021)
	*Microbacteriaceae/Microbacterium*	Plant growth promotion and protection	Ren et al. (2019); Gilbert et al. (2022)
	*Corynebactericeae/Rhodococcus*	Organic compounds degraders. Plant beneficial and pathogens	Savory et al. (2017); Kim et al. (2018)
*Bacillota*	*Bacillaceae/Bacillus*	Plant growth promotion and protection. Bioactive molecules synthesis	Rabbee et al. (2019); Andric et al. (2020); Blake et al. (2021)
	*Paenibacillaceae/Paenibacillus*	Plant growth promotion and protection. Antimicrobials synthesis	Grady et al. (2016)
*Bacteroidota*	*Flavobacteriaceae/Flavobacterium*	Gliding motility. Pollutant degradation. Pathogen (animal)	Shrivastava and Berg, (2015); Mishra et al. (2021)
*Pseudomonadota* (α class)	*Rhizobiaceae/Rhizobium/Agrobacterium/*	N fixation legume symbiosis. Plant DNA integration. Plant growth promotion	Barton et al. (2018); Ferguson et al. (2019); Harman & Uphoff (2019)
	*Sphingomonadaceae/Sphingomonas*	Plant growth promotion. Organic compounds degradation	Stolz (2014); Luo et al. [117]
	*Methylobacteriaceae/Methylobacterium*	Methylotrophy. Nitrate utilization. Anoxygenic phototrophy. Plant growth promotion	Ardanov et al. (2012); Knief et al. (2012); Iguchi et al. (2015); Macey et al. (2020); Alessa et al. (2021)
	*Devosiaceae/Devosia*	N fixation symbiosis	Rivas et al. (2002)
	*Bradyrhizobiaceae/Bradyrhizobium*	N fixation symbiosis	Jaiswal & Dakora (2019)
	*Azospirillaceae/Azospirillum*	N fixation. Plant growth promotion	Fukami et al. (2018)
	*Rhodobacteraceae/Paracoccus*	Methylotrophy	Czarnecki & Bartosik (2019)
*Pseudomonadota* (β class)	*Comamonadaceae/Polaromonas/Acidovorax/Variovorax/Pelomonas*	Aromatic compounds degradation. Plant sulfur supply. Nematode biocontrol	Pérez-Pantoja et al. (2012); Gahan & Schmalenberger, (2014); Topalovic et al. (2020)
	*Oxalobacteriaceae/Massilia*	Plant growth promotion and protection	Ofek et al. (2012); Liu et al. (2014)
	*Burkholderiaceae/(Para)Bulkholderia/Ralstonia*	Animal and plant pathogen. Plant growth promotion. Bioactive molecules synthesis. Aromatic compounds degradation	Pérez-Pantoja et al. (2012); Mannaa et al. 2019; Klaus et al. (2020); Xue et al. (2020)
*Pseudomonadota* (γ class)	*Pseudomonadaceae/Pseudomonas*	Metabolic versatility. Plant pathogen. Biocontrol. Plant growth promotion. Pollutant degradation. Bioactive molecules synthesis	Xin et al. (2018); Weimer et al. (2020); Zboralski and Filion (2020)
	*Erwiniaceae/Pantoea*	Plant pathogen. Biocontrol	Lahlali et al. (2022)
	*Xhantomonadaceae/Xhantomonas/ Stenotrophomonas/Lysobacter*	Plant pathogen. Plant growth promotion. Biocontrol	Puopolo et al. (2017); An et al. (2020); Tang et al. (2020)
	*Enterobacteriaceae/Enterobacter*	Plant growth promotion	Macedo-Raygoza et al. (2019); Roslan et al. (2020)
	*Moraxellaceae/Acinetobacter*	Plant growth promotion	Khanghahi et al. (2021)
*Non-bacterial*			
*Oomycota*	*Pythiaceae/Pythium*	Plant pathogen	Judelson and Ah-Fong (2019); Thambugala et al. (2020)
*Ascomycota*	*Mycosphaerellaceae*	Plant pathogen	Videira et al. (2017)
	*Davidiellaceae/Cladosporium*	Bioactive compounds	Salvatore et al. (2021)
	*Pleosporaceae/Alternaria*	Phytotoxins. Plant growth promotion	Thambugala et al. (2020). Wang et al. (2022)
	*Nectriaceae/Fusarium*	Plant pathogen	Rampersad (2020)
	*Trichocomaceae/Penicillium*	Versatile bioactive compounds synthesis. Plant growth promotion. Plant pathogen	Thambugala et al. (2020). Toghueo & Boyom, (2020); Bhatta (2022)
*Basidiomycota*	*Tremellaceae/Cryptococcus*	Human (mammals) pathogen	Bahn et al. (2020)
*Mucoronomycota*	*Mucoraceae/Rhizopus*	Animal and plant pathogen. Fermentation products	Gryganskyi et al. (2018)

*A selection of recent reviews, when available; if not, representative recent articles (only Open Access), are listed in the Supplementary List of References

## Microbial taxonomy in the different plant organs and compartments

### Root microbiota

#### Bacterial communities in the plant roots

Concerning root microbiota, Bulgarelli et al. [30] and Lundberg et al. [115] contributed by carefully defining the following root compartments: the endorhizosphere (the microbial. community found inside root tissues); the root-associated (the microbial community in close contact with the outer part of the roots), and the rhizosphere (the microbial community found in the proximity to roots), allowing finer distinctions among root microbial communities, and clearly distinguished them from bulk soil (not influenced by plants). However, procedures to obtain samples from these compartments are not strictly comparable in these and other studies (see subSect. "Experimental design, methods, and data analysis."), potentially explaining differences in microbial compositions of compartments.

Bulgarelli et al. [30] compared the bacterial communities in close contact with the roots of Arabidopsis. Using a slightly different operational definition of the plant-associated microbial community (endorhizospheric compartment), Lundberg et al. [115] found the same trend for plant or soil-associated communities, i.e., plants recruit a different subset of the surrounding soil microbiota. The main conclusion of these landmark studies is that *A. thaliana* root microbiota is composed of a core microbiome (for an in-depth analysis of this concept, see [172] of relatively few bacterial groups, mostly belonging to *Actinomycetota*, and the α, β, and γ *Pseudomonadota* classes (Fig. 1A), independently of the soil where the plants were collected from, the ecotype, or other factors. *Bacillota*, *Bacteroidota*, and a few other phyla were also found in these two and other reports (Table 2). Phylum relative abundances were different among these reports (Table 2), which is also observed at the family level in other studies (e.g., [101]. Just as representative cases (Table 2): α *Pseudomonadota* families *Caulobacteraceae* and *Rhizobiaceae* appear ranked in the top 3 of relative abundances, whereas *Sphingobacteraceae* shows different relative abundance positions; in β *Pseudomonadota* families, *Comamonadaceae* and *Oxalobacteraceae* are commonly found abundant, while *Burkholderiaceae* exhibits a variable ranking. Despite the expected differences in the bacterial community composition due to biological factors that may explain differences between these two studies (and several others), methodological factors also contribute to explaining such differences (sub-Sect. "Experimental design, methods, and data analysis.").

**Table 2:** Top relative abundances of bacterial endophyte's main phyla (A), and Pseudomonadota classes (B), determined by massive amplicon sequencing, reported in selected publications

Compartment phylum	Roots								Leaves				Flowers		Seeds
	Lu	Bu	Bo	S1	S2	S3	S4	Le	Bo	Ho	Ha	Ch	Boa*	Ma*,**	Jo***
A															
*Actinomycetota*	1º	2º	1º	3º	2º	3º	2º	1º	2º	3º	2º	3º	2º	2º	
*Pseudomonadota*	2º	1º	2º	1º	1º	1º	1º	2º	1º	1º	1º	1º	1º	1º	1º
*Bacteroidota*	3º	3º	3º	2º	3º	2º	3º	3º	3º	2º	4º		3º	4º	3º
*Bacillota*	4º	8º	4º	4º	6º		5º	4º	6º	6º	3º	2º	4º	3º	
*Cyanobacteriota*	5º									14º	5º				2º
*Cloroflexota*	6º				5º	4º	4º			4º					
*Acidobacteriota*	7º	6º	5º					5º	5º	7º	6º		5º		
*Armatimonadota*	8º		6º						4º	8º					
*Verrucomicrobiota*	9º	9º						6º		11º					
*Gemmatimonadota*	10º									9º			6º		
*Planctomycetota*		4º								12º					
*Candidate Division TM7*		5º	7º						7º	10º					
*Nitrospirota*		7º								13º					
*Deinococcota*		10º								5º					
*AD3*					4º										
*Euryarchaeota*											7º				
B															
*α Pseudomonadota*		4º	2º						1º		2º	2º	1º	2º	3º
β* Pseudomonadota*		2º	1º						2º		3º	1º	2º	1º	2º
γ *Pseudomonadota*		5º	3º						3º		1º	3º	3º	3º	1º

Lu et al. [115], Bu et al. [30], Bo et al. [23], S1-4: [169]: Le et al. [101], Ho et al. [78], Ha et al. [70], Ch et al. [40], Boa et al. [22], Ma et al. [126], Jo: J [85]. Numbers indicate cardinal top abundance position

^*^Floral samples are not necessarily endophytes

^**^A mistake in naming Pseudomonadota classes in this report (Fig. 3 vs. Additional file 1: Table S1)

^***^Bacillota are not abundant as indicated in Fig. 2 of that report (see Suppl. Mat. of that article. Data Sheet 1)

#### Non-bacterial communities in the plant roots

Bacterial communities are far more abundant (10–1000 times) than archaeal, fungal, and microeukaryotic communities in plant roots [50, 180]. However, these non-bacterial communities [72] play relevant roles in Arabidopsis performance (Fig. 1A). Bressan et al. [28] provided early evidence for the presence of archaeal species in this plant, and a low proportion of this domain was also observed in later studies [30, 115]. Furthermore, members of the *Thaumarchaeota* phylum have been described as relatively abundant, surpassing bacterial phyla such as *Nitrospirota* or *Bacillota* [184]. This becomes more relevant, considering that the PCR primer sets used in most studies are not primarily defined to detect archaeal species.

It should be kept in mind that Arabidopsis, like other *Brassicaceae*, does not harbor arbuscular mycorrhiza fungi (AMF) as most plant species, which means that its fungal community should have unique features. Members of the fungal phyla *Basidiomycota*, *Ascomycota*, and *Mucoromycota* or *Chytriomycota* can be found in significant numbers as part of the Arabidopsis root microbiota, but with variable predominance, based on relative abundances [180, 184, 191]. Urbina et al. [191] found *Archaeorhizomycetes* enriched in the rhizosphere and *Leotiomycetes* in the endosphere, both *Ascomycota* groups. Later, Fabiańska et al. [58] reported that eleven fungal orders were enriched in the rhizosphere, whereas four others were increased in the endosphere. Another study established that *Hypocreales,* *Pleorosporales*, and *Agaricales* were more abundant in roots compared to soils [60]. *Leotiomycetes* and *Sordariomycetes* have also been reported as abundant in seeds [50]. Interestingly, characteristic *Glomeromycota* mycorrhizal members were found in soils but not enriched in the plant compartments [58], which is consistent with the lack of mycorrhizal symbiosis in Arabidopsis. The basis for fungal endophytism in Arabidopsis has recently started to build up, with the finding that fungal functions like the secretion of cell wall-degrading enzymes and effector proteins are part of the repertoire of endophytic root mycobiota members [131].

A few recent studies provide helpful information regarding the presence of eukaryotic microorganisms other than fungi [72]. Using PCR primers targeting *Oomycota* and *Cercozoa*, Sapp et al. [165] found that *Oomycota* were not significantly different in soils compared to roots but discovered many diverse groups of *Cercozoa*, and some of them were preferentially found in roots. Durán et al. [50] described that *Pythium*, an *Oomycota* genus, was abundant in roots. Tkacz et al. [184] reported a scarcely described group, the microeukaryotes, finding that several metazoans are part of the root microbiota of the Arabidopsis holobiont. To help further studies with protists, a collection of nearly 80 *Cercozoa* isolates have been recently obtained from *A. thaliana* and is now available for future research for gnotobiotic studies [49], including the possibility of preparing Synthetic Communities (SynComs), i.e., simpler communities that mimic entire, complex communities ([109, 125, 199]). Concerning non-microbial groups closely interacting with microbial ones, Sikder et al. [174] have reported a vital nematode community in roots of Arabidopsis grown in soil, finding OTUs belonging to 75 species, from 32 families.

### Phyllosphere (leaf) microbiota

The microbiota of different compartments has been analyzed in the phyllosphere. For instance, the surface of the leaves, including epiphytes in the adaxial (upper surface) and abaxial (lower surface)] and the inner tissues (endophyllosphere). On the other hand, as Arabidopsis adult plants are small, shoot tissues are comparatively less relevant than leaves, so with a few exceptions (e.g., [60], the studies usually do no track shoot compartments (except for some targeting interactions among plant compartments, see Sect. "Microbial connections among plant compartments and their surroundings"), or they include them along with the leaves.

#### Bacterial communities in the phyllosphere

Phyllosphere microbiota has received less attention than root microbiota [31, 198]. Kniskern et al. [91] reported the presence of more than twenty different taxa in the culturable bacteria of endo- and the epiphyllosphere, including some well-known phytopathogens, presumably in an inactive state. Using Denaturing Gradient Gel Electrophoresis (DGGE) and clonal library approaches, a phylum bacterial composition, like the one reported for roots, was reported in the epiphyllosphere, including *Actinomycetota*, *Pseudomonadota,* and *Bacteroidota* [45]. Among *Pseudomonadota*, *Sphingomonas* were significantly abundant, whereas other families were found in detectable levels: *Rhizobiaceae*, *Oxalobacteraceae*, *Comamonadaceae*, and *Pseudomonadaceae* [45, 78]. Delmotte et al. [45] also applied a culture-independent metaproteomic approach, which showed the presence of a significant active population of other genera, mainly *Methylobacterium* -which use methanol coming from pectins-, along with several differences in relative abundances of different taxa. The relatively high abundance of the genus *Methylobacterium* in the epiphyllosphere of Arabidopsis was also reported using a ribosomal intergenic spacer analysis (RISA) and cultivation, defining *M. extorquens* as a key species [89, 90]. Key, or keystones, or hub species, play a fundamental role in the structure and functioning of the microbiota community [13, 72]. Interestingly, a relatively diverse bacterial community of anoxygenic photoautotrophs was detected in the phyllosphere, which might use sulfide provided by plants as a reductant [6]. This is consistent with the reported release of volatile sulfide compounds, mainly hydrogen sulfide, as a mediator of plant immunity and defense against microbial colonization in leaves (Vojtovic et al. 2020), thus providing a metabolic niche for anoxygenic photoautotrophy.

The leaf core community [23, 156], possesses as the main abundant phyla *Pseudomonadota*, followed by *Actinomycetota*, *Bacteroidota*, and then *Bacillota* (Fig. 1A, Table 2, [78]. As in roots, Arabidopsis leaf endophyte core community comprises the same significant phyla reported for most plant endophyte communities [70]. The main phyla found in a total phyllosphere bacterial community show a clear predominance of *Pseudomonadota* (> 90%) [10, 40]. Using fluorescence targeting, Remus-Enserman et al. [157]) found a heterogeneous distribution, with a significantly higher surface covered by bacteria on the abaxial side than on the adaxial one and concentrated in the borders of the stomata. Interestingly, essential levels of autofluorescence were found (Remus-Enserman et al., [157]), which may be explained by the presence of the abovementioned anoxygenic phototrophs [6]. An exometabolomic approach allowed finding a heterogeneous distribution of different organic compounds on the leaf surface which might explain the unequal distribution of phyllosphere bacterial community members [162]. Such distribution is probably due to differential amounts of sugars, nucleotides, and other molecules in the leaf source. Compared to the leaf surface, two to three orders of magnitude lower bacterial abundances (but with higher diversity) have been described in the endophyllosphere [40, 146]. These differences are explained mainly by the *Pseudomonadota* groups such as *Pseudomonas*, *Burkholderia*, and the *Actinomycetota* group *Kineosporia* [23].

#### Other microbial communities in the phyllosphere

As with roots, non-bacterial phyllosphere communities have received less attention [198]. Fungal members of *Ascomycota* and *Basidiomycota* are predominant in leaves (Fig. 1A, [20, 78]. The microeukaryotic community of the Arabidopsis phyllosphere has also been addressed, compared with other closely related plant species, but not much detail was provided, with the presence of some bacterivores and potential pathogens [148, 184]. No reports on archaeal members in the phyllosphere community have been published so far, except in Regalado et al. [155], where a tiny proportion of archaeal sequences was found.

### Seed and floral microbiota

Using the bacterial markers 16S rRNA and *gyrB*, and a fungal marker, Barret et al. [14] compared the seed microbiota among 20 *Brassicaceae*, including *A. thaliana*. Although bacterial and fungal richness exhibited significant variability, a common core was observed among all tested plant species, with *Pseudomonas*, *Pantoea*, *Xanthomonas*, and *Sphingomonas* as the main bacterial groups, and *Mycosphaerellaceae*, *Cladosporium*, and *Alternaria* among main fungal groups [14]. A comparison of the internal seed tissues and spermosphere microbiota (see a review of [137] of 17 angiosperm plant species, including Arabidopsis [85, 86], showed a predominance of *Pseudomonadota* and *Ascomycota*, with a significant proportion of common (present in more than 60% of plants) bacterial and fungal OTUs (Fig. 1A), with a few core (present in 100% of plants) bacteria (*Pantoea*, *Enterobacter*, *Pseudomonas*, *Bacillus*, among others), and one fungus, *Fusarium*.

There is only one report of the Arabidopsis floral microbiota [22], comparing Col-0 and two mutants of *CYP706A3* (P450) with decreased volatile terpene levels. They found about 350 OTUs belonging primarily to *Pseudomonadota* and *Actinomycetota,* with a few members of *Bacteroidota* and *Bacillota* (Fig. 1A) [22]. About 45 members changed their abundance in the cytochrome P450 mutant lines, suggesting a role of terpene volatiles in floral microbiota structure, specifically affecting taxa such as *Pseudomonas*.

## Microbial connections among plant compartments and their surroundings

One of the main factors influencing plant microbial communities is the physical connection, for example, between the surrounding soil and the plant roots, or the air and the leaves, and between compartments of the plant (rhizoplane and inner root parts, endorhizosphere and phyllosphere, etc.). These connections are responsible for the source (inoculum) of microbes that can reach and colonize a particular compartment defining its taxonomic composition and, therefore, playing a significant role in available microbial community functions and their interaction with the plant parts (see a summary on Fig. 1B).

### The soil as a microbial source in plants

Plants select their (core) microbiota (resident/transient) recruiting surrounding soil microbes, and, therefore, some microbial taxa are enriched in roots, whereas others are more abundant in the surrounding soil. Thus, the soil (both abiotic and biotic components) is the main factor explaining the root microbiota composition in Arabidopsis, and other plants [30, 47, 65, 115, 169, 183, 184, 194]. Typical phyla that are enriched in roots are *Pseudomonadota* and *Actinomycetota*, whereas *Acidobacteriota*, *Gemmatimonadota*, and *Verrucomicrobiota* are enriched in soils (Fig. 1A), with *Bacteroidota*, and *Bacillota* depending on the lower rank taxa that are analyzed [16, 30, 34, 36, 47, 60, 68, 101, 115, 186].

Concerning fungal and microeukaryotic members [191], and archaeal communities [180], the differences between surrounding soil and roots are less pronounced than those observed for bacteria. This could be explained because of a higher influence of the soil type and location on the eukaryotic communities compared to the plant factor [50, 159, 165, 184, 186]. Nevertheless, the effect of plants on fungal composition is reported to be significant [58], with increases in fungal diversity in roots [180] and endosphere [191]. *Hypocreales*, *Pleoroporales,* and *Agaricales* are enriched in roots, whereas *Filobasidiales* and *Mortierellales* are more abundant in soils than in roots [60].

Some studies differentiate the microbial community of the rhizosphere from the rhizoplane and the endorhizosphere compartments. Depending on the treatment of samples, the endorhizosphere community may be combined with or separated from the rhizoplane community (e.g., [30, 47, 50, 115, 169]. Several reports indicate that the endorhizosphere microbial communities are significantly different from those of the rhizosphere and, of course, those of bulk soils [101, 115, 159, 169]. Endorhizospheric communities are the less diverse, although the prominent members correspond to enriched taxa found both in the rhizosphere and the soil compartments [30, 50, 115]. Thiergart et al. [186] studied the root microbiota from Arabidopsis populations grown in 17 locations in Europe (a latitudinal transect) for three years. They found that bacterial communities were more affected by soil (edaphic factors) than the location (climatic factors), whereas the contrary was observed for fungal and oomycotal communities. The main drivers for endorhizosphere communities (quantity and availability) were reserve and available P, and manganese for bacteria and fungi, respectively [186]. In summary, the surrounding bulk soil is the rhizosphere's primary source of bacterial microbiota. In turn, this compartment is the source for the inner parts of the plant (Fig. 1B, [31].

### The air as a microbial source in plants

To address the effect of the “"air” factor (Fig. 1B), Maignien et al. [124] conducted carefully controlled tests under greenhouse conditions. They found that the phyllosphere composition was defined by early succession events driven by a combination of a plant selective (filtering) process that recruits specific taxa from the air present in low abundances, such as *Variovorax*, *Rhodococcus*, *Methylobacterium*, with stochastic effects mainly reflected in the variability of abundances of these taxa, related to the position of plant individuals in the greenhouse.

### Microbial connections among plant compartments

Using a SynCom approach, Bai et al. [10] reported the effect of spray inoculation of SynComs from leaves, roots, and soil, in leaves and in soil planted or not with Arabidopsis. They reported that the established phyllosphere community resulted differently when the leaves SynCom was inoculated in the leaves or in the soil, the latter containing not only soil sources but also air and dust. When plants were exposed to the three SynComs, the root compartment was colonized by the root and soil SynComs members, indicating that leaf isolates were less adapted to the root environment. Furthermore, Hou et al. [79, 80], nicely demonstrated a microbiota-roots-shoots bidirectional connection in plant response to suboptimal light conditions. Part of these effects were provoked by ectopic colonization of leaves from root-inoculated bacterial commensals [79].

The contribution of the seed microbiota (inner and external tissues) to other compartments was studied by growing 17 plant species, including Arabidopsis, under sterile conditions and comparing them with those grown on natural soil [85, 86]. They reported significant input of seed microorganisms to root and shoot microbiota in most plants, including Arabidopsis, and to a lower degree in the rhizosphere microbiota (Fig. 1B) [85]. This provides strong evidence for vertical transmission controlling the microbiota of seedlings.

One of the most unexplored connections is that of soil to flowers. Massoni et al. [126] nicely compared the phyllosphere and floral microbiota in plants exposed only to soil microorganisms under closed lab conditions or under open-air garden conditions. They showed that about 25% of the bacterial taxa found in the floral microbiota originated from soil microorganisms and therefore were internally transported (Fig. 1B), irrespective of the growth conditions. These bacteria represented more than 75% of the relative abundances, and 24 out of 28 bacteria that marked floral microbiota belonged to *Burkholderiaceae*. It is worth mentioning that in most cases, this family is found at low, and more commonly, very low levels in the roots and phyllosphere, which reflects the strong selection followed by the plant, making this finding even more remarkable. A member of this family, *Paraburkholderia phytofirmans* PsJN, has been proposed as a source of vertical transmission to seeds when applied to flowers of different plants [134], and is able to affect floral transition in Arabidopsis [151].

## Methodological issues in plant microbiota studies

### Germ-free studies

Germ-free plant models represent abnormal conditions [145]. Currently, the presence of seed microbiota in plants is undoubted [173]. An important part of publications on Arabidopsis microbiota indicates germ-free conditions lacking a proper experimental confirmation. Sterile seeds could be explained by producing seeds for many generations under laboratory conditions with low microbial loads (agar, artificial soil mixtures, etc.). Thus, it is perfectly conceivable, but not systematically addressed yet, that *A. thaliana* seed batches (and therefore, the derived plantlets and adult individuals) would harbor different (both in composition and structure) microbial communities, depending on the time and conditions of maintenance in the lab. Truyens et al. [190] clearly demonstrated significant losses in the richness and abundance of the bacterial community after several generations of *A. thaliana* seeds grown in bacteria-poor substrates. Therefore, part of the knowledge of the biology of this plant model is not correctly addressed if this natural microbial source is not considered.

### Experimental design, methods, and data analysis

A large variety of experimental choices is observed regarding plant age/development, ecotypes (accessions), sampling procedures (including specific plant microzones), targeted 16S rRNA sequence sites, PCR primer sequences (Table 3), PCR conditions, and DNA sequence amplification technologies. Thirteen different primer pair sequence combinations targeting 16S rRNA have been used, although those targeting V3-V4 (several combinations of), V4, and V5-V7 sequences, represent 23, 20, and 34% of reports, respectively (Table 3). In a few cases, a couple of PCR primer sequences were compared, and significant differences were found (e.g., [115, 186]). For instance, the primer pair targeting the V5-V7 region showed less bias than the primer pair amplifying the V3-V4 sequence [186]. In addition, there are differences in DNA sequence data analysis, data bioinformatics pipelines, statistical packages, and alignment and taxonomical databases. For instance, bioinformatic removal of non-target sequences is not always declared or detailed. Although OTUs are used in more than 85% of reports, amplicon/amplified/actual sequence variants have been increasingly utilized in the last 5-years publications. Statistical procedures such as normalization or rarefaction are dissimilarly stated or detailed. All these differences, some of them noted in previous publications (e.g., [169]) should be considered to compare reports. Proposals and guidelines to improve and correct biases and misinterpretations in sequence data analysis have been published, e.g., Sczyrba et al. [171], and Lucaciu et al. [113].

**Table 3:** rRNA sequence variations in next generation sequencing data acquisition to study Arabidopsis microbiota

rRNA gene target	Primer pair sequences	Publications that use it. (Declared drawbacks/biases/modifications)
V1-V2	27F/338R	[183] (1)
V1-V3	27F: 5′-AGAGTTTGATYMTGGCTCAG-3′ 533R: 5′-TTACCGCGGCTGCTGGC-3′	[39]
V3-V4	314F: 5′-CCTACGGGNGGCWGCAG-3′ 805R: 5′-GACTACHVGGGTATCTAATCC-3′	[111, 205] (2) [207], (3) [106],
V3-V4	338F: 5′-ACTCCTACGGGAGGCAGCA-3 806R: 5′-GGACTACHVGGGTWTCTAAT-3′	[60, 110, 112, 154, 163]
V3-V4	341F: 5′-CCTAYGGGRBGCASCAG-3′ 806R: 5′-GGACTACNNGGGTATCTAAT-3′ 341F: 5′-CCTACGGGAGGCAGCAG-3′ 806R: 5′-GGACTACHVGGGTWTCTAAT-3′	[2] (1) [117], (2) [108], (2) [203], (2) [52, 186],
V3-V4	341F: 5′-CCTAYGGGRBGCASCAG-3′ Uni806R:5′-GGACTACNNGGGTATCTAAT-3′	[204] (4)
V3-V4	S-D-Bact-0341-b-S-17: 5′-CCTACGGGNGGCWGCAG-3′ S-D-Bact-0785-a-A-21: 5′-GACTACHVGGGTATCTAATCC-3′	[98, 174]
V3-V5	341F/907R:	[156] (1)
V3-V5	479F: 5′-CAGCMGCYGCNGTAANAC-3′ 888R: 5′-CCGYCAATTCMTTTRAGT-3′	[130]
V4	515F: 5 ‘-GTGYCAGCMGCCGCGGTAA-3′ 806R:5′-GGACTACNVGGGTWTCTAAT-3′	[142] (1) [68], (1) [14], (1) [101], (1) [143], (1) [36], (1) [57], (1) [54, 155, 170, 184, 194], (1) [85], (1)
V4-V5	518F/926R	[82] (1) [46], (1)
V4-V6	518F: 5′-CCAGCAGCYGCGGTAAN-3′ 1046R: 5′-CGACRRCCATGCANCACCT-3′	[124]
V5-V6	784F: 5′-RGGATTAGATACCC 1064R: 5′-CGACRRCCATGCANCACCT	[178]
V5-V7	799F**2**: 5´-AACMGGATTAGATACCC**G**G-3´ 1193R: 5′-ACGCATCCCCACCTTCCTC-3′ 799F: 5′-AACMGGATTAGATACCCKG-3′ 1193(2)R: 5′ACGTCATCCCCACCTTCC-3′	[30] (5), (6) [23], (7) [2, 78, 169], (1) [47, 158, 190], (1) [22, 25, 50, 81, 159], (1) [16, 40, 69, 186, 201], (1) [202], (1) [87, 126, 146], (1) [51, 52],
V5-V8	799F: 5′-AACMGGATTAGATACCCKG-3′ 1392R: 5′-ACGGGCGGTGTGTRC-3′	[19]
V5-V8	803F: 5′-ATTAGATACCCTGGTAGTC-3′ 1392wR: 5′-ACGGGCGGTGWGTRC-3′	[10, 33–35]
V5-V8	804F: 5′-ATTAGATACCCDRGTAGT-3′ 926F: 5′-AAACTYAAAKGAATTGACGG-3′′ 1392R: 5′-ACGGGCGGTGTGRC-3′	[115]
V6-V8	1114F: 5′-GCAACGAGCGCAACCC-3′ 1392R: 5′-ACGGGCGGTGTGRC-3′	[115]
V8	1114F-1392R	[101] (1)

(1) PCR primer sequences not provided in that text

(2) The sequence of primer 314F has some differences from other published

(3) Primer 341F corresponds to 314F

(4) Modified broad 341F and Uni806R

(5) 799F2 is a single nucleotide modification (bolded) of F799 to avoid amplification of 18S rRNA

(6) 799F2 produced an overrepresentation of *Chloroflexota* sequences that was computationally removed from data analysis

(7) 1193R designed by authors

(8) 1392R and not 1392wR is indicated as in Carvalhais et al. [33]

This variety of choices may explain rather gross differences in the relative abundance of higher-rank taxa groups (Table 1, [10, 30, 54, 111, 115, 170, 177, 178, 180, 204, 205], and more evidently, in lower rank taxa groups, and less abundant taxa, e.g., the presence of archaeal taxa [184]. In a few cases, inconsistencies were detected and even corrected, such as the presence of an unexpected abundance of *Chloroflexota* [30], in other cases, PCR bias was previously known, and caution analyzing results was applied [148, 165, 184], or an internal validation using more than one approach was performed [47, 101, 115]). The hidden effects of experimental and data analysis variability may lead to wrong conclusions, and the need to systematize protocols and bioinformatic and statistics approaches should be addressed by the Arabidopsis research community.

In this context, most reports use 16S rRNA as a molecular marker for bacteria. Few exceptions use *gyrB*, targeting the β subunit of the bacterial gyrase [14, 15], which allows the identification of the sequences to the species level and does not have a variation of copy number per genome. Although Bartoli et al. [15] also reported *Pseudomonadota* being the most abundant phylum in the main compartments, the estimated abundance (> 80%) exceeded by far that reported using the 16S rRNA marker. The use of both *gyrB,* and 16S rRNA, allowed to detect important changes in community composition and relative abundances associated with the developmental stages of plants [14]. Use of PhyloChip also indicated that *Pseudomonadota* was the predominant phylum in *A. thaliana* rhizosphere, exceeding by two the relative abundance of *Bacillota* and *Bacteroidota*, and by ten those of *Cyanobacteriota*, *Actinomycetota*, and *Verrucomicrobiota* [18], a pattern quite different of that reported using Next Generation Sequences approaches. On the other hand, a comparison of two plant culture systems (pots and rhizoboxes) added another layer of fluctuations as several growth parameters of Arabidopsis plants differed significatively [130]. In this context, Kremer et al. [95] have proposed a plant growth system/protocol for microbiota research.

Regalado et al. [155] compared rRNA gene amplicon analyses with metagenome shotgun sequencing to study Arabidopsis leaf microbiota. Although with the shotgun strategy, they found a similar pattern as reported in other publications, they determined different interactions between the ten main family taxa (positive turned negative or neutral) when both methodologies were compared. The discrepancies are produced because microbial abundances can be related to plant DNA abundances in the shotgun procedure, whereas amplicon analyses are usually based on microbial relative abundances. Microbial absolute abundances should be based on plant material determinations (e.g., [51, 202]. Although primarily designed to avoid/minimize the amplification of Arabidopsis nontarget DNA [2], blocking oligos can also be used to estimate bacterial loads and determine absolute abundances [128]. Blocking oligos has proved to be effective in avoiding the amplification of non-target DNA from plastids, and microeukaryotes, improving the bacterial sequence depth [128]. An improved approach to both better quantification of microbial loads and description of community compositions has been recently reported [116].

## Arabidopsis features that shape its microbial communities

The plant holobiont sustains reciprocal regulations between microbes and the plant [192]. This section focuses on the plant as a selective recruiter of microbiota to enhance plant fitness under a suite of environmental conditions (Fig. 2A).

### The role of the plant ecotype modulating the plant microbiota

In terms of the ecotype, most of the studies used Col-0 (~ 65%), a lower proportion (~ 25%), more than 15 different accessions, or wild populations (~ 15%), and a few did not mention ecotype. Concerning ecotype effects, early work using Terminal Restriction Fragment Length Polymorphism, RISA, DGGE, and high-performance liquid chromatography to track plant root exudates (PRE) composition differences evidenced that bacterial communities differed among ecotypes [132, 133]; dissimilarities related to the distinct PRE compositions of each ecotype. Then, ecotype effects on a small subset of unique OTUs were reported [30, 115]. Later, Horton et al. [78] tested 196 worldwide accessions and used Genome-Wide Associations Studies; they found that richness was affected by host genetic variation and was associated with *loci* involved in virus responses, trichome branching, and morphogenesis. Other comparisons of microbial community structures across accessions revealed the main keystone role of *Comamonadaceae* in bacterial communities and *Articulospora* in fungal communities [13]. Network analysis of the data from different accession communities showed more positive than negative interactions, with fungal members being relevant in shaping leaves and roots communities [20]. A well-defined effect of nine different accessions has also been demonstrated for SynComs [24], whereas other Arabidopsis accessions impact *Pseudomonas fluorescens* populations depending on each specific ecotype [68]. Differences among Col-0, Ws-O, and Ksk-1 ecotypes were found for bacterial, fungal, and oomycetal microbiota components [2]. Cold tolerance Arabidopsis accessions also showed changes in their microbiota composition [57]. In addition to host genotype effects on the Arabidopsis microbiota, ploidy levels, which vary in wild and managed populations, also affected microbial communities, as recently reported [129, 149].

### Plant circadian cycle effects on the microbiota

Staley et al. [178] demonstrated changes in the bacterial community (at several taxonomic levels) depending on the phase of the day. Hubbard et al. [82] confirmed the role of the circadian cycle in the root bacterial communities, comparing the Ws ecotype with two circadian cycle mutants (short, 20 h and large, 28 h). In a more detailed analysis, Lu et al. [112] studied transcriptomic and metabolomic changes in Col-0 and two other acyclic mutants, finding changes in plant gene expression and PRE components in both mutants comparing the wild-type. Multiple regression analysis performed with data of PRE components and relative abundances showed a strong correlation which agrees with previously reported effects of the circadian clock in PRE composition, partially explaining changes in the bacterial community [178]. Also, mutant plants in LHY (a transcription factor involved in circadian rhythm) displayed rhizosphere microbiota changes, especially in fungal groups involved in plant health [138].

### Effects of the developmental stage on the plant microbiota

Chaparro et al. [39] compared bacterial communities at seedling, vegetative, bolting, and flowering stages, and found that the seedling stage community mainly differed from the other three, with relatively few gross changes in composition throughout the life cycle. Such differences were mainly explained by variations in the relative abundances of a few genera: among others, *Streptomyces*, *Solibacter, Flexibacter*, and *Leptolyngbya*, which might be due to changes in PRE composition. A comparison of the phyllosphere microbiota in bolting, flowering, and maturation showed a set of 40 genera that were shared among these three stages; 27 genera belonging to *Pseudomonadota*, six to *Actinomycetota*, five to *Bacteroidota,* and two to *Bacillota* [106]. Enriched members associated with flowering were, among others, *Azospirillum*, *Methylobacterium, Paracoccus*, and *Nocardioides*.

Host developmental effects on natural bacterial assemblages have been followed in field samples during all the stages of the annual cycle of Arabidopsis [16]. They found that endophyte bacterial community compositions from roots, shoots, and leaves were driven by the same set of commensals that were not especially prevalent but whose relative abundances changed among compartments. Similar conclusions were obtained when young and old leaves were compared [19].

Community composition changes at early seed germination and emergence have been reported for bacteria and fungi from several plants, mostly *Brassicaceae*, including *A. thaliana* [14]. Contrasting seeds with germination and emergence (seedlings) microbiota, they found essential changes at the emergence stage, characterized by an apparent decrease in bacterial and fungal richness. However, a note of caution on the interpretation of the developmental stage's effects on microbial communities should be considered, as Dibner et al. [46] reported that elapsed time (and resulting microbial succession) has more potent effects on communities than on the specific stage.

### Role of PRE and specific plant metabolites

Among the effects that plants exert on their microbial communities, those played by simple molecules, alone or in complex mixtures as in PRE [11], have received considerable attention [31]. Early work on Arabidopsis fungal communities showed that biomass and phylotype richness, measured by internally transcribed sequences profiling, was significantly affected by in vitro prepared PRE [29]. An ABC-transporter Arabidopsis mutant with an altered PRE composition produced changes (mainly decreases in abundances) in bacterial and fungal communities [8]. Further evidence on the role of PRE in shaping rhizobacterial communities was suggested when PRE effects from eight Arabidopsis ecotypes were compared [133]. In addition, the role of PRE influencing microbial communities has been proposed to explain differences between bacterial communities from the root and the rhizospheric compartment, with specific sub-groups of α and β *Pseudomonadota* classes being differentially active [67].

Small PRE molecules are classified as amino acids, sugars, organic acids, and phenolics [7]. Considering this, Badri et al. [9] tested different PRE compound mixtures on unplanted soil communities, finding changes in relative abundances at low taxonomical levels rather than in higher ones. It is worth mentioning that the unidentified compounds present in PRE are still a majority fraction and that the assignation of PRE compounds to the phenolics or organic acids category used in some reports is not free of confusion. An inspection of compounds listed as phenolics (aromatic ring compounds with at least one hydroxyl substitution) shows that this category included fatty acids, non-aromatic organic acids, alcohols, aliphatic hydroxy acids, and N-containing compounds, being actual phenolics only one-tenth of compounds listed as such. Recent reports have shown that aromatics are significantly lower than sugars, amino acids, and lipids [77], with a relative abundance of lipids and lipids-like components higher than 40% [112]. It is out of the question that further progress in analytical tools is needed to fully understand PRE composition [139].

Phytohormone changes in plant physiology produce alterations in PRE composition and, therefore, in microbial communities, as reported for jasmonic acid (JA) [33, 34]. Furthermore, using a triple mutant in Dicer-like genes involved in small RNAs biogenesis, Kaushal et al. [87] found an altered PRE composition, which can partially explain the observed modifications in the root microbial community.

The nutrient status also produces changes in PRE. Herrera Paredes et al. [77] used the SynCom approach to test plants exposed to different available P levels and found that the ability of some SynCom members to grow on PRE components was not necessarily a good proxy of their performance on plant growth tests. On the contrary, binary plant-bacteria interactions (positive, neutral, negative) were better predictors of SynCom’s effects on plants. Using the ^13^C DNA Stable Isotope Probing technique, Worsley et al. [203] demonstrated that several *Pseudomonadota* genera incorporated ^13^C from PRE components when Arabidopsis is grown on compost.

The role of specific groups of plant-derived compounds on microbial communities has been addressed [84, 181]. Early work using DGGE and ^13^CO_2_ labeling reported that glucosinolates, typical *Brassicaceae* secondary metabolites that are precursors of biocidal compounds protecting plants from pathogens, produced significant changes in the Arabidopsis fungal and the α *Pseudomonadota* class (*Rhizobiaceae*) communities [28]. The use of nine glucosinolate plant mutants confirmed extensive effects on root microbiota, with several bacterial and fungal taxa being enriched in the mutants compared with the wild type [98].

The role of a PRE component, scopoletin (a coumarin siderophore and antimicrobial compound), in shaping Arabidopsis microbiota has been demonstrated by comparing the microbial communities from the wild type, and a scopoletin synthesis mutant [180]. The effect of coumarins has been further explored using several plant biosynthetic mutants and the addition of specific coumarins, using SynComs and gnotobiotic cultivation, for better elucidation of direct from indirect effects [196]. Fraxetin, another coumarin synthesized directly from scopoletin, that has antimicrobial effects and affects iron availability, also modified the Arabidopsis root microbiota, differentially affecting relative abundances of lower-rank taxa such as *Burkholderiaceae*, *Rhizobiaceae*, and *Streptomycetaceae* [69]. Recently, flavonoids were also related to changes in the microbiota diversity in Arabidopsis [74].

The role of PRE macromolecules [11], has received no attention in Arabidopsis. It should be noted that released mucilage can harbor protein and DNA, as well as small carbon source molecules that would contribute to shaping rhizosphere microbiota [166].

### Effects of plant responses to abiotic stress on the plant microbiota

The effects on the microbiota of Arabidopsis exposed to abiotic stress have started to be studied. Reports on heavy metal pollutants (cadmium, [188], cold [57], drought [117], and nutrient limitations: iron [69, 180, 196] and phosphate [58, 60, 77, 159] are now available.

Although there are several reports on the effects in Arabidopsis of both inorganic and organic pollutants (e.g., [42, 102], to date, only a few reports address the effects of contaminants on Arabidopsis microbiota [108, 188, 190, 207]. Truyens et al. [188] used a culture-dependent approach and found shifts in seed endophyte composition when plants were exposed to Cd for several generations. More recently, the effects of glyphosate on Arabidopsis growth and its phyllosphere microbial community [154] were mainly detected when this herbicide was applied in carbon nanotubes [88]. Effects on microbiota (rhizosphere diversity, community composition, and interspecies interaction) of Arabidopsis and *Nicotiana benthamiana* of per- and polyfluoroalkyl pollutants have been recently reported [110].

The effect of low temperatures on Arabidopsis phyllosphere microbiota was analyzed in different accessions during cold acclimation [57]. Although the main phyllosphere phyla (*Pseudomonadota*, *Actinomycetota*, *Bacillota*, and *Bacteroidota*) did not show alterations between cold-acclimated and non-acclimated accessions, differences were found at lower taxonomical ranks. This might be explained by different plant gene expression patterns and plant biomolecule synthesis upon cold acclimation [57].

The connection between microbiota and the impact of drought conditions was initially addressed by Zolla et al. [208], determining that 41 genera were enriched in the Arabidopsis soil slurry,  a significant part of these genera belonging to the Arabidopsis core microbiota [30, 115]. In this context, it has also been reported that bacterial communities significantly changed under drought [117], observing a decrease in the diversities of the the rhizoplane and rhizosphere communities [204]. Using a four-member consortium that protected Arabidopsis from drought conditions, Yang et al. [204] showed significant re-shaping of the microbial assembly in water-stressed plants in such a way that inoculated plants exhibited a bacterial community resembling that of plants grown under watered conditions. Regarding salt stress, chronic exposure to salt produced changes in the bacterial leaf community, with altered relative abundances in members of *Actinomycetota*, *Bacillota*, and *Pseudomonadota* [19]. They reported that community assembly resulted from three interacting factors: the leaf age, the abscisic acid (ABA) phytohormone biosynthesis (as part of the abiotic stress response), and the function of *PBS3*, a signaling component of the defense phytohormone SA. *PBS3* plays a crucial role in regulating the trade-off between biotic and abiotic stresses.

The effect of the responses of plants to suboptimal light conditions was recently addressed using a three-kingdom SynCom, composed of 183 bacteria, 24 fungi, and seven oomycetes, and several combinations of them [79]. Suboptimal light produced significant changes in the composition of the root bacterial (but not the fungal and oomycetal) community.

The effects of iron and phosphorous (phosphate) availability on Arabidopsis microbiota have received consideration. Applying a β diversity comparison, iron depletion was the second factor differentiating Arabidopsis microbial community after the bulk soil versus the rhizosphere soil factor [180]. Relative abundances of 21 genera changed (mostly belonging to *Pseudomonadota* and *Bacillota*) at different iron levels. Additionally, under full iron conditions, no changes in microbial composition were observed, but under iron-limiting conditions, the presence of iron mobilizing coumarin (see [181]) drastically reduced *Pseudomonadaceae*, among other taxa [196]. Bodenhausen et al. [25] reported that OTUs belonging to *Burkholderiales*, *Bdellovibrionales*, and *Rhodocyclales* showed increased relative abundances at low P levels. Diverse microbial communities are also found in the wild-type and phosphate starvation response (PSR) Arabidopsis mutants at low taxonomic rank levels [36, 77]. Finkel et al. [60] reported changes in roots and shoots fungal and bacterial communities regarding P availability and PSR mutations. Further evidence of the effects of PSR genotype and P levels on the fungal community has been reported by Fabiańska et al. [58], identifying, for example, that full-P conditions increase the relative abundance of *Microascales* and *Olpidiales* and the decrease of *Helotiales* and *Xylariales*. However, the link among the phosphorous levels, microbiota (fungal communities), and plant responses, is more complex and is not completely understood yet (Macía-Vicente et al. 2022).

### Effects of plant responses to biotic stress on the plant microbiota

Plants face several biotic stresses, with the effect of phytopathogens the more frequently addressed. The inoculation on leaves with the biotrophic fungal pathogen *Hyaloperonospora arabidopsidis* produced detectable changes in *A. thaliana* rhizosphere, these changes were not produced by the necrotrophic pathogen *Botrytis cinerea* or salicylic acid (SA) and methyl jasmonic acid applications [18]. A soil-mediated effect of this biotrophic pathogen led to protecting newly planted Arabidopsis, which may be due to changes in the rhizosphere microbiota [18]. Another fungal pathogen, *Golovinomyces orontii*, causing the foliar disease powdery mildew, produced changes in the leaf (but not roots) fungal and bacterial communities [51].

It has been reported that inoculation of leaves with the phytopathogen *Pseudomonas syringae* pv. *tomato* in Arabidopsis grown in successive transfers to the same soil produced changes in both bulk and rhizospheric soil microbiota [205]. These changes were partially associated with an increase in the relative abundance of a few initially low-abundance taxa: *Fictibacillus* and *Roseiflexus*, and a decrease in the relative abundance of *Sphingomonas* [18]. Arabidopsis plants infected with this phytopathogen showed disease suppression by soil legacy in a phenomenon probably related to the relative abundance of some PRE components [205]. Recently, a synergistic effect between a fungal endophyte and the resident bacteria microbiota against a fungal hemibiotrophic pathogen was reported (Madhli et al. 2022).

The role of different hormones related to Induced Systemic Resistance (ISR) and Systemic Acquired Resistance (SAR) in shaping plant microbiomes has also been studied [33, 48, 75, 91] and [101]. Kudjordjie et al. [98] reported the effects on root microbial community assembly of seven Arabidopsis mutants in defense signaling molecules: SA, ethylene (ET), JA, ABA, and fatty acid desaturase linked to the regulation of SA and JA pathways. The microbiota of these mutants showed changes, in different degrees, in richness, α and β diversity, of both bacterial and fungal taxa, supporting the role played by plant defense in shaping the wild-type microbial community. Strigolactones, plant hormones involved in plant development and chemical communication during biotic interactions (as those involved in plant—AMF relations), affected the fungal but not the bacterial diversity when the microbiota of the Arabidopsis wild type and a mutant with impaired strigolactones synthesis were compared [35]. Impaired lines of Arabidopsis in JA, SA, ET, and strigolactones synthesis showed changes in nematode taxa abundances, they produced changes in the fungal and bacterial community when the plant was grown in natural soil conditions [174, 175]. Resistance and susceptibility to the pathogen *Fusarium oxysporum* f.sp *mathioli* resulted from a combination of plant ecotype and metabolome and robustness of the microbial assembly [99].

The effects of other plant protection processes have also been tested. The role of the cuticle, the first mechanical barrier to colonizing the phyllosphere, was studied using plant mutants possessing different cuticle wax compositions. Reisberg et al. [156] found a role in shaping core, resident, and transient bacterial phyllosphere communities, the latter the more numerous. Similarly, changes in the phyllosphere microbiota were reported in two cuticle *A. thaliana* mutants resistant to the phytopathogen *B. cinerea* [158].

As indicated above, changes in the leaf metabolome composition have been reported when pathogenic and non-pathogenic species colonize plants [162]. The protective role of specific compounds, in this case, triterpenes involved in immunity and antibiosis, has also been explored in roots [81]. Several triterpene biosynthetic mutants exhibited different microbial communities.

A quadruple mutant in the innate immune and vesicle trafficking pathways showed a severe alteration in leaf endophytes (but not in the total leaf community) associated with chlorosis and necrotic effects [40]. Using endophyte SynComs from the mutant and wild-type plants, the authors showed that when the mutant SynCom was infiltrated in wild-type leaves, the plant developed chlorosis and necrotic effects, otherwise, when a wild type SynCom was inoculated in the mutant plant, the damaged leaf tissue effects diminished. Therefore, the dysbiosis caused by these mutations increases the abundance of bacteria producing leaf damage that is, in turn, were constrained by the microbiota of the healthy plant. Changes in the endophyllosphere have also been reported in several immune system mutants using gnotobiotic growth systems and SynComs [146]. Such changes allowed increased colonization of opportunist pathogens, especially a *Xanthomonas* sp. strain, that produce disease and affect plant growth. The role of commensal pseudomonas strains protecting against pathogens, promoting host response has been recently reported [176, 200].

The role of the leaf microbiota on protection against *P. syringae* pv. *tomato* DC3000 has been studied in individual isolates of the SynCom At-LSPHERE, and in selected ten-and three- members combinations [195]. Direct microbe-to-microbe interactions and indirect plant-mediated processes are possible mechanisms behind phytopathogen protection [195]. When selected commensals of this At-LSPHERE were tested in mono associations with Arabidopsis, a defined small set of plant genes was up-regulated [123]. The authors defined this set of genes as part of a general non-self-response, helping to distinguish pathogenic from non-pathogenic bacteria. This non-self-response was differentially triggered by these commensals, with variable intensities across close or distantly related strains [56, 123, 182]. This non-self-response is involved in the interaction between the immune system and microbiota, with the former shaping the host-microbial community and the latter influencing the immune response and defense mechanisms. For further details on the interaction of innate immunity and microbiota, see the review of Hacquard et al. [66]. Interestingly, the role of epigenetic regulation in shaping plant microbial communities has started to be addressed. For instance, a dysfunction in the histone demethylase IBM1 produced strong effects in the assembly of root microbiota through the regulation of SA-genes regulating plant immunity [118].

## Reciprocal influence of plants and their microbiota

The focus of this section is to consider the microbiota as a facilitator, i.e., providing additional genes to the host to adjust to local environmental conditions [192] (Fig. 2B). The taxonomical composition of communities should reflect the functions they provide to the plant. Those abundant phyla would represent a broader range of tasks than those less abundant. Such seems to be in Arabidopsis, where the predominant *Pseudomonadota* is far more functionally diverse than the other less abundant phyla [10]. In this context, Lau and Lennon (2012), correction in 2021) performed a multigeneration selection experiment that manipulated the soil-moisture environment of replicated plant populations and their associated microbial communities. They found a strong effect of the microbe's evolutionary history on plant fitness responses to this abiotic stress.

Notably, soil microbiota control plant functions such as flowering time [142, 143]. This phenomenon was further studied with selected Col-0 microbiota from early and late flowering times plants, transferred to other Arabidopsis ecotypes, and the crucifer *Brassica rapa* [142]. Early or late flowering root microbiota accelerates or delays the flowering of inoculated plants. Few low-abundance families were dissimilarly present in early (*Xhantomonadaceae* and *Pseudomonadaceae*) or late (*Iamiaceae*, *Alcaligenaceae*, and *Corynebacteriaceae*) flowering microbial communities. The relative abundance of the main phyla reported associated with the Arabidopsis core microbiota (see subSect. "Root microbiota"., Table 1, and [143] were found altered in early flowering microbiota with *Bacillota* (31%) being more abundant than *Actinomycetota* (22%), *Pseudomonadota* (17%), and *Bacteroidota* (16%). In a later work, delayed flowering in the wild type and early flowering in a photosynthetic *A. thaliana* mutant (*pgr5*) was observed in plants grown on a sterile growth medium inoculated with a slurry containing Arabidopsis root microbiota obtained from previous growth rounds [111].

A survey for bacterial genes that are involved in adaptation to plants has been reported searching genomes from isolates of two Arabidopsis ecotypes (including Col-0), maize and poplar, and a comparison with nearly 3500 other genomes from soil isolates and isolates non-adapted to plants, to define plant-associated genes [104]. Plant-associated genomes possess more “Carbon and Transport” genes and fewer “Mobile Elements” genes than non-plant-associated genomes, with an essential overlap in genes from plant-associated and root-associated and between non-plant-associated and soil genome isolates [105]. Interestingly, plant-associated genomes harbor bacterial protein domains mimicking plant domains that are shared with fungi and oomycetes associated with plants [104], which may help microorganisms camouflage.

In another proof of the reciprocal influence between the plant and its microbiota, Salas-González et al. [163] reported that alterations in genes controlling diffusion barriers, involved in mineral nutrient balance in the root endodermis, produce changes in roots and shoots microbiota assembly. Root microbiota controls endodermic differentiation (mainly suberin deposition) through repression of ABA transcriptional response. Microbial effects were observed in plants grown on agar and soil, with a 41-members SynCom, and in binary tests as well [163]. Significantly, changes in suberin deposition produced by microorganisms when plants were exposed to nutrient stress led to systemic effects in the ionomes of the plants. Impact on the rhizosphere microbial community in mutants in components of a signaling pathway of endodermal root organization (ERK1 and TIC) and lignin and suberin deposition have also been reported using 206-members AtRSPHERE-based SynCom [54]. In another layer of interaction among plant immune response and plant microbiota, root commensals that suppress the root growth inhibition response also significantly affect the expression of subsets of Arabidopsis genes, increasing the expression of genes related to root development, nutrient transport and decreasing some genes related to immune response and detoxification [119]. Such differences in gene expression are not observed when plants are inoculated with non-suppressive commensals.

### Microbial interactions in the plant microbiota

Interactions among different microbial groups of the Arabidopsis microbiota are crucial to understanding its dynamics and the effects on the host [72] (Fig. 2B). Seminal work shed some light on the role of protozoa in the bacterial community [96, 97, 161]. The Arabidopsis growth promotion, as observed in other species, takes place by the inoculation of the protozoon *Acanthamoeba castellanii*. In other words, plant growth promotion is increased when bacteria and protozoan are both present [96]. The effects of *A. castellanii* on the Arabidopsis bacterial community were selective as β *Pseudomonadota* class and *Bacillota* decreased their relative abundance. In contrast, *Actinomycetota*, *Nitrospirota*, *Verrucomicrobiota,* and *Planctomycetota* increased their relative abundance, measured by DGGE clone library and sequencing [161].

A three-kingdom component (bacteria, fungi, and oomycetes) interaction has been investigated in the Arabidopsis phyllosphere, exploring different ecotypes and abiotic conditions [2]. Using network analysis, the authors identified microbial hubs, key species [13, 27, 193], whose presence/absence severely affects microbial community structure. They further studied some of these microbial hubs showing that the biotroph pathogen oomycete *Albugo* affected the colonization of both bacterial epiphytes and endophytes (decreasing α diversity); the *Basidiomycota* yeast *Dioszegia* interacted negatively with phyllosphere bacteria, and that a bacterial hub *Comamonadaceae* genus positively interacted with other bacterial groups [2]. Mainly inhibitory interactions between phyllosphere bacterial taxa have been reported, which are explained by the antimicrobial effects of different biosynthetic gene clusters detected in the phyllosphere microbiota members [76]. Relatively less abundant *Bacillales* and *Pseudomonadales* microbiota members caused the most inhibitory effects on bacterial target groups, which rarely include closely related bacterial taxa [76]. *Actinomycetota* species involved in negative interactions in the phyllosphere microbiota have been recently identified using SynCom [168].

Based on reported data [20], He et al. [73] performed network analyses [193] of microbe-to-microbe (bacteria and fungi) interactions (mutualism, antagonism, aggression, and altruism) in the root microbiota from 179 Arabidopsis accessions. They found that bacteria have more connections in mutualism and altruism interactions, whereas fungi were more critical in antagonism and aggression. They, as performed in Agler et al. [2], searched for microbial hubs, reporting 59, mostly belonging to *Pseudomonadota* (21), *Actinomycetota* (12), and *Ascomycota* (20), with antagonism (26) and altruism (24) having far more hubs than mutualism (6) and aggression (3).

Bartoli et al. [15] addressed the interaction between the microbiota, essentially non-pathogenic, with the pathobiota fraction. They tested 163 native *A. thaliana* bacterial populations from different ecologically contrasting habitats in France and found that at low or high α diversity microbiota levels, the pathobiota α diversity levels were low, with higher pathobiota α diversity levels found at intermediate levels of microbiota diversity (the invasion paradox). Tracking of the microbiota species that lead to the pathobiota changes determined a dozen OTUs, unclassified, with a few exceptions, among the latter *Pseudomonas moraviensis* [15].

Interactions between prokaryotic and eukaryotic members help maintain Arabidopsis health, as judged by several SynCom inter-kingdom combinations [50, 202], being positive interactions predominant between bacterial groups, whereas adverse effects dominate fungal/oomycetal interactions with bacteria. SynCom with fungal/oomycetal members negatively affected plant health, while bacterial SynCom positively acted on Arabidopsis health. *Variovorax* and *Acidovorax,* were the best to rescue the plant from the adverse effects of eukaryotic members [50].

An exciting phenomenon revealing multilevel interactions among root bacteria was reported in Arabidopsis [61]. A 185-member SynCom produced severe root growth inhibition. All the *Variovorax* genus strains from this SynCom could suppress the root growth inhibition exerted by the whole SynCom, subsets, and unique strains. Stimulatingly, the mechanism underlying this phenomenon is related with an interference in the signaling of auxin [44].

Carlström et al. [32] studied the effect of bacterial groups’ arrival times from the same taxa or individual strains in structuring the bacterial community. They used a 62-phyllosphere members SynCom and found that assembly is historically contingent (time of arrival), subjected to priority, and resistant to late arrivals. Through single strain drop-out tests (a single strain is initially absent in the community but added later) and causal network analysis, they detected keystone strains (*Microbacterium* and *Rhodococcus*, and *Sphingomonas* and *Rhizobium*) and the predominance of negative (competition) direct or indirect interactions [32]. Priority effects in root community assembly have also been reported, as non-native species were less abundant when introduced after an initial inoculation of commensals [201]. This phenomenon needs a living root and some components of PRE, does not depend on immune response functions, and correlates with the strength of commensal invasiveness [119].

### Plant growth promoting rhizobacteria effects on Arabidopsis microbiota

Plant Growth Promoting Rhizobacteria (PGPR) has a tremendous potential to increase crop productivity through the improvement of plant nutrition as well as plant protection [59]. Despite the more than 250 articles on PGPR and Arabidopsis, relatively few of them study the effects of PGPR in the Arabidopsis microbiota. Haney et al. [68] tested the impact on the whole microbiota of the inoculation of one PGPR *P. fluorescens* strain, finding relatively few changes, with some enrichments in *Bacillota* and losses in *Bacteroidota*. This observation raises an interesting point, largely unexplored in this kind of report, about the actual promotion or protection being a direct effect of inoculated PGPR or a consequence of the activation or depletion of pre-existing populations in the microbiota.

Using combinations of N-fixing, non-N-fixing, symbiotic and non-symbiotic species isolated from other plants and soils, Garrido-Oter et al. [64] reported plant gene expression associated explicitly with an interfered immune system response. It should be noted that such decreased immune response is found when some *Rhizobiales* establish an N-fixing, nodule-forming symbiosis [94], which is not a possibility in Arabidopsis. However, Arabidopsis-endophyte *Ensifer meliloti* interaction provides N to the plant [5]. Therefore, it is possible to think that *Rhizobiales* are normal inhabitants in Arabidopsis that may modulate the immune response. Evidence for a role as a PGPR for some *Rhizobiales* is also reported [64], whose colonization process may be benefited by this immune system interference. Root immune system interference has also been shown for bacterial commensals [182]. They found that about one-third of 35 members Syncoms [36] suppressed microbe-associated molecular patterns triggered immunity. Arabidopsis growth promotion by a bacterial/fungal/oomycetal SynCom is also observed but abolished in immunocompromised plants [202]. The fungal component (dysbiosis due to fungal load) of this SynCom is responsible for altered plant growth promotion in the immune response mutants.

In a different context, hydroponic culture tests of the effects of 96 Arabidopsis root isolates in the maintenance of a *Bacillus subtilis* PGPR indicated that a relatively small fraction of them, not phylogenetically related, helped this PGPR to survive in binary co-cultures [55]. Among the helpers, a *Variovorax* isolate better performed with *B*. *subtilis* and the other two *B*. *amyloquefaciens* PGPR. A recent report explores the effects of another PGPR, *Sphingomonas* sp. Cra2 [117], finding that the inoculant increased the relative abundances of members *Burkholderiales* and *Pseudomonadales*, to whom several PGPR species have been reported [103, 114, 147, 152, 167, 187, 206, 209], Orellana et al. 2022). This suggests that benefic effects may be produced by changes in the root microbiota that are part of the promotion effects of the added strain stimulating PGPR species present in the root system [117]. A drought-protecting four members consortium that re-shapes Arabidopsis microbiota also has plant growth promotion effects [204]. In addition, the presence of the siderophore scopoletin strongly affects the Arabidopsis microbial community with the enhanced presence of genera having PGPR and metal or N uptake properties [180].

## Arabidopsis microbiota in comparison with other plant species

Several studies compare the Arabidopsis microbiota with other plant species. A first report compared Arabidopsis phyllosphere with those of soybean, rice, and clover [45, 198]. Using DGGE, the authors found that α diversity was higher in Arabidopsis and that each plant species has a particular and stable in-time bacterial profile, although sharing about 70% of species with similar relative abundances. One of the main taxa found in Arabidopsis phyllosphere, *Methylobacterium*, was further explored by comparing this community to that found in several other plant species, including *M. truncatula*. The authors found that this community primarily depended on the site, but also on the plant species [90]. Anoxygenic phototrophs found in the phyllosphere of Arabidopsis have also been found in clover, rice, and soybean [6].

An in-depth comparison of the microbiota from root/rhizosphere compartments among Arabidopsis, Arabidopsis species (*A. halleri* and *A. lyrata*), and the *Brassicaceae* relative *Cardamine hirsuta*, showed similar bacterial communities, being the main groups *Actinomycetotales, Burkholderiales*, *Flavobacteriales*, *Rhizobiales*, and *Sphingomonadales*, with the differences attributable to specific taxa [169]. They also found that about one-half of the diversity is shared among plants, with the other half explaining adaptative differences such as resistance to metals (*A. halleri*), or being perennial (*A. halleri*, and *A. lyrata*). A similar degree of sharing among high-rank taxa was reported between Arabidopsis and the legume *L. japonicus*, with the shared taxa showing different abundances [201]. These authors also showed that a clear host preference for root commensals was observed in *A. thaliana* and *A. lyrata*.

Similarities among microbial communities of closely related *Brassicaceae* species (*Draba verna*, *C. hirsuta*, and *C. pratensis*) are not always found. Poch et al. (2017) used PCR primers to detect *Cercozoa* in the phyllosphere. They found that shared groups were only one-fifth of the sequences and that *A. thaliana* was the more distant species. However, a common group of bacterial and fungal species has been reported in seeds from 20 *Brassicaceae*, including *A. thaliana*, and several non-*Brassicaceae* species [14]. In a meta-analysis, Hacquard et al. [65] compared the composition of the root bacterial community of Arabidopsis and its relatives [169], with that of other plants such as soybean, wheat, and cucumber. This work found that root plant communities were more similar among them than with those of human gut and other animals, where *Bacteroidota* and *Bacillota* dominate, but closer to the fish gut where *Pseudomonadota* were abundant, as in plants.

A comparison among *A. thaliana* (Col-0), *A. alpina,* and *C. hirsuta* showed that the factor species only explain 7–10% of the variability among bacterial communities, indicating microbiota closeness which includes nearly 20 shared families [47]. Interestingly, shared core microbiota members can also be found when not-so-close plant species (poplar, maize, and *Brassicaceae*) are considered [105]. In another context, a minor fraction of the shared Arabidopsis taxa with rice and wheat was affected in Arabidopsis triterpene biosynthesis mutants, indicating that triterpene effects are species-specific and contribute to the recruitment of Arabidopsis microbiota from soil [81].

The comparison of bacterial community successions after three generations in model plants (*A. thaliana* as a non-legume, *M. truncatula* as a legume, and *Brachypodium distachyon* as a monocot cereal) and crops (*B. rappa*, *Pisum sativum*, and *Triticum aestivum*), allowed to determine that the plant factor is, after soil source, the more important factor shaping these communities [183]. This critical role of the plant species was more significant with bacterial communities than with fungal communities, and with evident differences found in Arabidopsis, including more archaeal groups than in the other three species. The shared OTUs taxa belonged to relatively rare groups (*Verrucomicrobiota* > *Chloroflexota* > *Thaumarchaeota* >  > *Actinomycetota* = *Acidobacteriota*), with the scarce presence of *Pseudomonadota* OTUs [184]. Bacterial and fungal community comparisons among Arabidopsis with eight other phylogenetically diverse plant species thriving in the same semi-natural grassland habitat, showed that endorhizosphere bacterial communities were substantially shared [170]. In contrast, fungal communities, especially that of the rhizosphere compartment were more variable among these plants, with Arabidopsis sharing only around 10% of fungal OTUs, as has been also reported by Bergelson et al. [20]. Among the nine plants, Arabidopsis rhizosphere bacterial community was the one which have less OTUs that change their relative abundance with respect to the bulk soil, indicating a comparatively lower rhizosphere effect [170], as reported also by Tkacz et al. (2015, 2020).

Comparison of the bacterial community from duckweed, a phylogenetically distant monocot aquatic plant, with the microbiota of Arabidopsis phyllosphere indicated a similar assemblage, with *Pseudomonadota*, *Bacteroidota*, *Bacillota*, and *Actinomycetota* as main phyla, although the latter being significantly less abundant in duckweed [1]. A general similar root-associated bacterial community was reported in a comparative study between Arabidopsis and Petunia, a plant that establishes interactions with AMF, with β- and γ *Pseudomonadota*, and *Bacteroidota* as more abundant taxa, and with Petunia exhibiting higher richness and diversity than Arabidopsis [25]. This overall similitude was not reflected when plants were exposed to different P levels, as changes in relative abundances were found for different bacterial groups in both plants. Going forward, Durán et al. [52] contrasted the microbial community of Arabidopsis with that of a taxonomically distant photosynthetic organism, the subaerial green model alga *Chlamydomonas reinhardtii*. The authors reported taxonomically similar communities, a core microbiota, with relatively abundant phyla, as indicated in Table 2, between the phycosphere and the plant roots, with larger abundances in *Bacillota* and lower in *Actinomycetota*, in the alga compared to the plant.

In a different approach, Bieker et al. [21] compared the leaf microbiota of Arabidopsis with that of the annual weed *Ambrosia artemisiifolia* and contrasted the metagenomic profiles of ancient samples (collected up to 180 years ago and available in herbariums). The authors reported different leaf metagenomic profiles, and variations between ancient and modern samples for both species, with the differential presence of specific fungal sequences in ancient samples attributable to contamination during preparation and storage. Ancient samples of Arabidopsis showed sequences reported in modern leaf samples such as *Pseudomonas putida* and *Albugo* spp.

## Concluding remarks and future research needs

Several aspects of the Arabidopsis microbiota were discussed in detail in the previous sections, revealing how intertwined each of the factors that modulate these complex biological relationships are (Fig. 2B). Despite that, Arabidopsis allows us to unveil the intricacies of the mechanisms behind host-microbe interactions and their effects on plant holobiont fitness. Arabidopsis provides grounds to answer most, if not all, questions on molecular plant–microbe interactions posed in a recent survey [71]. The depth, critical and exhaustive analysis provided here is, so far, the most comprehensive review of the Arabidopsis microbiota, and brings light to the understanding of such fundamental biological interactions. There are some limitations in this model as it does not establish AMF symbiotic relations nor is a nodulating plant [4, 166], which, in turn, is a useful tool as a non-symbiotic plant control. Interestingly, it is not clear, yet which evolutionary and genetic limitations constrain these symbiotic interactions to specific taxa.

Then, there is a need to move forward (Fig. 2C) and consider the Arabidopsis microbiota/microbiome as a complex system, where the outputs are not the sum of the parts, and multifactorial approaches should be addressed. For instance, the plant-life history traits, epigenome, evolutionary, ecological, and climate (as recently addressed by [53] aspects, and concomitant multiple layers of the abiotic and biotic aspects of the environment should be included in future research. Networking mapping analysis [107] and multi-genome metabolic modeling [127] are some of the ways to move forward in this issue. This will foster our ability to predict the effects of a particular plant-microbiota interaction and to engineer the use of microbiomes for more sustainable agriculture. More specifically, what is clear is the lack of omics approaches to understand Arabidopsis–microbe interactions, other than metagenomics analysis based only on the 16S sequence marker, i.e., taxonomy-based analysis. There are, comparatively, only a few reports so far, addressing functional metagenomics (metatranscriptomics, metaproteomics, metametabolomics) in Arabidopsis [104]. They are clearly required as early proposed by Bakker et al. [12]. A recent approach for metaproteomic analyses of rhizosphere microorganisms has been reported [164]. It is quite possible that technical problems based on low biomass, which are not observed in bigger plant species such as cucumber, soybean, and wheat [65], explain this Arabidopsis drawback, which should be surpassed soon as more sensitive/resolutive methods and technologies come available. Gnotobiotic plant systems may help in this direction as allow analysis of microbiota functions under easily controlled experimental conditions [120].

An effort of the Arabidopsis research community [144] to set the basis to make Arabidopsis (and other plants) microbiota studies more systematically comparable [31] is clearly required to provide a strong basis for analysis between different reports for a single species and comparisons among diverse species. Such a systematic methodological approach will be required to perform finer studies like comparisons of microbiota at different root lengths/sites (root tip, intermediate and mature roots, and secondary growth roots) and their connection with PRE rhizodeposition components [31]. Lateral position/border cells studies on rhizosphere microbiota effects are also enlightened by Sasse et al. [166], which in turn is linked to the role of macromolecular components of PRE, mucilage, and other components derived from sloughed cells [31].

Other studies that would benefit from having a systematic experimental approach will be comparisons of the adaxial and abaxial sides of leaf microbiota, microbial loads of seeds, and those addressing the rare and semi-rare bacterial community [180]. Also, consensus operational procedures would be helpful in dealing with poorly studied issues such as the circadian cycle [178] and seasonal effects [15, 31]. In addition, seed microbiota functions should be considered: i.e. ecology, developmental stages, shaping of the core microbiota, vertical transmission [41, 137, 189], and seed-to-seed cycle [15]. Finally, an unexpected lack of reports on the effects of Arabidopsis microbiota/microbiome in issues such as nitrogen limitation [94], or plant–insect (nematode)-microbe interactions, is clearly a call for new studies. In addition, there is a complete lack of reports on Arabidopsis virome. Even though it is suggested that viruses, not only those that cause diseases, also have commensal and mutualistic interactions, helping plants to overcome abiotic stresses [150], and shaping plant ecology and evolution [160]. In turn, bacteriophages and temperate phages can also contribute to modify the ecology and evolution of plant-associated microbial communities and should be considered in some analyses [93, 153]. Then, Arabidopsis may also be a helpful plant model for studying plant virome in-depth (Fig. 2C).

## Supplementary Information

**Additional file 1**. Supplementary List of References (cited in Table 1).

## Data Availability

Not applicable.

## Declarations

### Content for publication

The authors give the consent for publication to the Publisher.
