# Supplementary material for: The Arabidopsis holobiont: a (re)source of insights to understand the amazing world of plant–microbe interactions
Source: Environ Microbiome. 2023 Feb 17;18:9. doi: 10.1186/s40793-023-00466-0 (PMC9938593; doi:10.1186/s40793-023-00466-0)
Supplement: Supplementary file 1 — Additional file 1. Supplementary List of References (cited in Table 1). [file 40793_2023_466_MOESM1_ESM.docx]

Supplementary Material

The Arabidopsis holobiont: a (re)source of insights to understand the amazing world of plant-microbe interactions.

Submitted to Environmental Microbiome

Poupin, M.J.^1,2,3^, Ledger, T.^1,2,3^, Roselló-Móra, R.^4^, González, B.^1,2,3*^.

Affiliations:

^1^Laboratorio de Bioingeniería, Facultad de Ingeniería y Ciencias, Universidad Adolfo Ibáñez, Santiago, Chile.

^2^Center of Applied Ecology and Sustainability (CAPES). Santiago. Chile.

^3^Millennium Nucleus for the Development of Super Adaptable Plants (MN-SAP), Santiago, Chile.

^4^Marine Microbiology Group, Department of Animal and Microbial Biodiversity. Mediterranean Institute for Advanced Studies (IMEDEA UIB-CSIC), Illes Balears, Mallorca, Spain.

^*^Corresponding author: Facultad de Ingeniería y Ciencias. Universidad Adolfo Ibáñez. Postal code: 7941169. Santiago, Chile. Phone: 56-2-3311619. Fax: 56-2-3311906. e-mail: [bernardo.gonzalez@uai.cl](mailto:bernardo.gonzalez@uai.cl).

**Supplementary List of References (cited in Table I).**

Alessa, O., Ogura, Y., Fujitani, Y., Takami, H., Hayashi, T., Sahin, N., and Tani, A. (2021). Comprehensive comparative genomics and phenotyping of *Methylobacterium* species. Front. Microbiol. 12:740610. <https://doi.org/10.3389/fmicb.2021.740610>.

An, S.-Q., Potnis, N., Dow, M., Vorhölter, F.-J., He, Y.-Q., Becker, A., Teper, D., Li, Y., Wang, N., Bleris, L., and Tang, J.-L. (2020). Mechanistics insights into host adaptation, virulence and epidemiology of the phytopathogen *Xanthomonas*. FEMS Rev. 44:1-32. <https://doi.org/10.1093/femsre/fuz024>.

Andric, S., Meyer, T., and Ongena, M. (2020). *Bacillus* responses to plant-associated fungal and bacterial communities. Front. Microbiol. 11:1350. <https://doi.org/10.3389/fmicb.2020.01350>.

Ardanov, P., Sessitch, A., Häggman, H., Kozyrovska, N., and Pirttilä, A.M. (2012). *Methylobacterium*-induced endophyte community changes correspond with protection of plants against pathogen attack. PLoS ONE 7:e46802. <https://doi.org/10.1371/journal.pone.0046802>.

Bahn, Y.-S., Sun, S., Heitman, J., and Lin, X. (2020). Microbe profile: *Cryptococcus neoformans* species complex. Microbiology. 166:797-799. <https://doi.org/10.1099/mic.0.000973>.

Barton, I.S., Fuqua, C., and Platt, T.G. (2018). Ecological and evolutionary dynamics of a model facultative pathogen: *Agrobacterium* and crown gall disease of plants. Environ. Microbiol. 20:16-29. <https://doi.org/10.1111/1462-2920.13976>.

Bhatta, U.K. (2022). Alternative management approaches of citrus diseases caused by *Penicillium digitatum* (green mold) and *Penicillium italicum* (blue mold). Front. Plant. Sci. 12:833328. <https://doi.org/10.3389/fpls.2021.833328>.

Blake, C., Norgaard Christensen, M., and Kovács, Á.T. (2021). Molecular aspects of plant growth promotion and protection by *Bacillus subtilis*. Mol. Plant Microbe-Interact. 34:15-25. <https://doi.org/10.1094/MPMI-08-20-0225-CR>.

Czarnecki, J., and Bartosik, D. (2019). Diversity of methylotrophy pathways in the genus *Paracoccus* (*Alphaproteobacteria*). Curr. Issues Mol. Biol. 33:117-131. <https://doi.org/10.21775/cimb.033.117>.

Ferguson, B.J., Mens, C., Hastwell, A.H., Zhang, M., Su, H., Jones C.H., Chu, X., and Gresshoff, P.M. (2019). Legume nodulation: The host controls the party. Plant Cell Environ. 42:41-51. <https://doi.org/10.1111/pce.13348>.

Fukami, J., Cerezini, P., and Hungria, M. (2018). *Azospirillum*: benefits that go far beyond biological nitrogen fixation. AMB Expr. 8:73. <https://doi.org/10.1186/s13568-018-0608-1>.

Gahan, J., and Schmalenberger, A. (2014). The role of bacteria and mycorrhiza in plant sulfur supply. Front Plant Sci. 5:723. <https://doi.org/10.3389/fpls.2014.00723>.

Gilbert, S., Poulev, A., Chrisler, W., Acosta, K., Orr, G., Lebeis, S., and Lam, E. (2022). Auxin producing bacteria from duckweeds have different colonization patterns and effects on plant morphology. Plants. 11:721. <https://doi.org/10.3390/plants11060721>.

Grady, E.N., MacDonald, J., Liu, L., Richman, A., and Yuan, Z.-C. (2016). Current knowledge and perspectives of *Paenibacillus*: a review. Microb. Cell Fact. 15:203. <https://doi.org/10.1186/s12934-016-0603-7>.

Gryganskyi, A.P., Golan, J., Dolatabadi, S., Mondo, S., Robb, S., Idnurm, A., Muszewska, A., Steczkiewicz, K., Masonjones, S., Liao, H.-L., Gajdeczka, M.T., Anike, F., Vuek, A., Anishchenko, I.M., Voigt, K., Sybren de Hoog., G., Smith, M.E., Heitman, J., Vilgalys, R., and Stajich, J.E. (2018). Phylogenetic and phylogenomic definition of *Rhizopus* species. G3 Genes. Genomes. Genetics. 8:2007-2018. <https://doi.org/10.1534/g3.118.200235>.

Harman, G.E., and Uphoff, N. (2019). Symbiotic root-endophytic soil microbes improve crop productivity and provide environmental benefits. Scientifica. 2019. <https://doi.org/10.1155/2019/9106395>.

Iguchi, H., Yurimoto, H., and Sakai, Y. (2015). Interactions of methylotrophs with plants and other heterotrophic bacteria. Microorganisms. 3:137-151. <https://doi.org/10.3390/microorganisms3020137>.

Jaiswal, S., and Dakora, F.D. (2019). Widespread distribution of highly adapted *Bradyrhizobium* species nodulating diverse legumes in Africa. Front. Microbiol. 10:310. <https://doi.org/10.3389/fmicb.2019.00310>.

Judelson, H.S., and Ah-Fong, A.M.V. (2019). Exchanges at the plant-oomycete interface that influence disease. Plant Physiol. 179:1198-1211. <https://doi.org/10.1104/pp.18.00979>.

Khanghahi, M.Y., Strafella, S., Allegretta, I., and Crecchio, C. (2021). Isolation of bacteria with potential plant-promoting traits and optimization of their growth conditions. Curr. Microbiol. 78:464-478. <https://doi.org/10.1007/s00284-020-02303-w>.

Kim, D., Choi, K.Y., Yoo, M., Zylstra, G.J., and Kim, E. (2018). Biotechnological potential of *Rhodococcus* biodegradative pathways. J. Microbiol. Biotechnol. 28:1037-1051. <https://doi.org/10.4014/jmb.1712.12017>.

Klaus, J.R., Coulon, P.M.L., Koirala, P., Seyedsayamdost, M.R., Déziel, E., and Chandler, J.R. (2020). Secondary metabolites from the *Burkholderia pseudomallei* complex: structure, ecology, and evolution. J. Ind. Microbiol. Biotechnol. 47:877-887. <https://doi.org/10.1007/s10295-020-02317-0>.

Knief, C., Delmotte, N., Chaffron, S., Stark, M., Innerebner, G., Wassmann, R., von Mering, C., and Vorholt, J.A. (2012). Metaproteogenomic analysis of microbial communities in the phyllosphere and rhizosphere of rice. ISME J. 6:1378-1390. <https://doi.org/10.1038/ismej.2011.192>

Lahlali, R., Ezrari, S., Radouane, N., Kenfaoui, J., Esmaeel, Q., El Hamss, H., Belabess, Z., and Barka, E.A. (2022). Biological control of plant pathogens: a global perspective. Microorganisms, 10:596. <https://doi.org/10.3390/microorganisms10030596>.

Liu, J., Liu, S., Sun, K., Sheng, Y., Gu, Y., and Gao, Y. (2014). Colonization on root surface by a phenanthrene-degrading endophytic bacterium and its application for reducing plant phenanthrene contamination. PLos ONE 9:e108249. <https://doi.org/10.1371/journal.pone.0108249>.

Luo, Y., Wang, F., Huang, Y., Zhou, M., Gao, J., Yan, T., Sheng, H., and An, L. (2019). *Sphingomonas* sp. Cra20 increases plant growth rate and alters rhizosphere microbial community structure of *Arabidopsis thaliana* under drought stress. Front. Microbiol. 10:1221. <https://doi.org/10.3389/fmicb.2019.01221>.

Macedo-Raygoza, G.M., Valdez-Salas, B., Prado, F.M., Prieto, K.R., Yamaguchi, L.F., Kato, M.J., Canto-Canché, B.B., Carrillo-Beltrán, M., di Mascio, P., White, J.F., and Beltrán-Garcia, M.J. (2019). *Enterobacter cloacae*, an endophyte that establishes a nutrient-transfer symbiosis with banana plants and protects against the black sigatoka pathogen. Front. Microbiol. <https://doi.org/10.3389/fmicb.2019.00804>.

Macey, M.C., Pratscher, J., Crombie, A.T., and Murrell, J.C. (2020). Impact of plants on the diversity and activity of methylotrophs in soil. Microbiome. 8:31. <https://doi.org/10.1186/s40168-020-00801-4>.

Mannaa, M., Park, I., and Seo, Y.-S. (2019). Genomic features and insights into the taxonomy, virulence, and benevolence of plant-associated *Burkholderia* species. Int. J. Mol. Sci. 20:121. <https://doi.org/10.3390/ijms20010121>.

Mishra, S., Lin, Z., Pang, S., Zhang, W., Bhatt, P., and Chen, S. (2021). Recent advanced technologies for the characterization of xenobiotic-degrading microorganisms and microbial communities. Front. Bioeng. Biotechnol. 9:632059. <https://doi.org/10.3389/fbioe.2021.632059>.

Ofek, M., Hadar, Y., and Minz, D. (2012). Ecology of root colonizing *Massilia* (Oxalobacteraceae). PLoS ONE 7:e40117. <https://doi.org/10.1371/journal.pone.0040117>.

Okazaki, K., Tsurumaru, H., Hashimoto, M., Takahashi, H., Okubo, T., Ohwada, T., Minamisawa, K., and Ikeda, S. (2021). Community analysis-based screening of plant growth-promoting bacteria for sugar beet. Microbes Environ. 36:2021. <https://doi.org/10.1264/jsme2.ME20137>.

Olanrewaju, O.S., and Babalola, O.O. (2019). *Streptomyces*: implications and interactions in plant growth promotion. Appl. Microbiol. Biotechnol. 103:1179-1188. <https://doi.org/10.1007/s00253-018-09577-y>.

Pérez-Pantoja, D., Donoso R, Agulló L, Córdova M, Seeger M, Pieper DH, and González, B. (2012). Genomic analysis of the potential for aromatic compounds biodegradation in *Burkholderiales*. Environmental Microbiology. 14:1091–1117. <https://doi.org/10.1111/j.1462-2920.2011.02613.x>.

Puopolo, G., Tomada, S., and Pertot, I. (2017). The impact of omics era on the knowledge and use of *Lysobacter* species to control phytopathogenic micro-organisms. J. Appl. Microbiol. 124:15-27. <https://doi.org/10.1111/jam.13607>.

Rabbee, M.F., Ali, M.S., Choi, J., Hwang, B.S., Jeong, S.C., and Baek, K.-H. (2019). *Bacillus velezensis:* A valuable member of bioactive molecules within plant microbiomes. Molecules. 24:1046. <https://doi.org/10.3390/molecules24061046>.

Rampersad, S.N. (2020). Pathogenomics and management of *Fusarium* diseases in plants. Pathogens. 9:340. <https://doi.org/10.3390/pathogens9050340>.

Ren, X.-M., Guo, S.-J., Tian, W., Chen, Y., Han, H., Chen, E., Li, B.-L., Li, Y.-Y., and Chen, Z.-J. (2019). Effects of plant growth-promoting bacteria (PGPB) inoculation on the growth, antioxidant activity, Cu uptake, and bacterial community structure of rape (*Brassica napus* L.) grown in Cu-contaminated agricultural soil. Front. Microbiol. 10:1455. <https://doi.org/10.3389/fmicb.2019.01455>.

Rivas, R., Velázquez, E., Willems, A., Vizcaíno, N., Subba-Rao, N.S., Mateos, P.F., Gillis, M., Dazzo, F.B., and Martínez-Molina, E. (2002). A new species of *Devosia* that forms a unique nitrogen-fixing root-nodule symbiosis with the aquatic legume *Neptunia natans* (L.f.) Druce. Appl. Environ. Microbiol. 68:5217-5222. <https://doi.org/10.1128/AEM.68.11.5217-5222.2002>.

Roslan, M.A.M., Zulkifli, N.N., Sobri, Z.M., Zuan, A.T.K., Cheak, S.C., and Abdul Rahman, N.A. (2020). Seed biopriming with P- and K-solubilizing *Enterobacter hormaechei* sp. improves the early vegetative growth and the P and K uptake of okra (*Abelmoschus esculentus*) seedling. PLoS One 15:e0232860. <https://doi.org/10.1371/journal.pone.0232860>.

Salvatore, M.M., Andolfi, A., and Nicoletti, R. (2021). The genus *Cladosporium*: a rich source of diverse and bioactive natural compounds. Molecules. 26:3959. <https://doi.org/10.3390/molecules26133959>.

Savory, E.A., Fuller, S.L., Wiesberg, A.J., Thomas, W.J., Gordon, M.I., Stevens, D.M., Creason, A.L., Belcher, M.S., Serdani, M., Wiseman, M.S., Grünwald, N.J., Putnam, M.L., and Chang, J.H. (2017). Evolutionary transitions between beneficial and phytopathogenic *Rhodococcus* challenge disease management. eLife. 6:e30925. <https://doi.org/10.7554/eLife.30925>.

Shrivastava, A., and Berg, H.C. (2015). Towards a model for *Flavobacterium* gliding. Curr. Opin. Microbiol. 28:93-97. <https://doi.org/10.1016/j.mib.2015.07.018>.

Stolz, A. (2014). Degradative plasmids from sphingomonads. FEMS Microbiol. Lett. 350:9-19. <https://doi.org/10.1111/1574-6968.12283>.

Tang, A., Haruna, A.O., Majid, N.M.A., and Jalloh, M.B. (2020). Potential PGPR properties of cellulolytic, nitrogen-fixing, phosphate-solubilizing bacteria in rehabilitated tropical forest soil. Microorganisms. 8:442. <https://doi.org/10.3390/microorganisms8030442>.

Thambugala, K.M., Daranagama, D.A., Phillips, A.J.L., Kannangara, S.D., and Promputtha, I. (2020). Fungi vs. fungi in biocontrol: an overview of fungal antagonists applied against fungal plant pathogens. Front. Cell. Infect. Microbiol. 10:606923. <https://doi.org/10.3389/fcimb.2020.604923>.

Toghueo, R.M.K., and Boyom, F.F. (2020). Endophytic *Penicillium* species and their agricultural, biotechnological, and pharmaceutical applications. 3 Biotech. 10:107. <https://doi.org/10.1007/s13205-020-2081-1>.

Topalovic, O., Hussain, M., and Heuer, H. (2020). Plants and associated soil microbiota cooperatively suppress plant-parasitic nematodes. Front. Microbiol. 11:313. <https://doi.org/10.3389/fmicb.2020.00313>.

Videira, S.I.R., Groenewald, J.Z., Nakashima, C., Braun, U., Barreto, R.W., de Witt, P.J.G.M., and Crous, P.W. (2017). *Mycosphaerellaceae* – chaos or clarity? Stud. Mycol. 87:257-421. <https://doi.org/10.1016/j.simyco.2017.09.003>.

Wang, H., Guo, Y., Luo, Z., Gao, L., Li, R., Zhang, Y., Kalaji, H.M., Qiang, S., and Chen, S. (2022). Recent advances in *Alternaria* phytotoxins: a review of their occurrence, structure, bioactivity, and biosynthesis. J. Fungi. 8:168. <https://doi.org/10.3390/jof8020168>.

Weimer, A., Kohlstedt, M., Volke, D.C., Nikel, P.I., and Wittmann. (2020). Industrial biotechnology of *Pseudomonas putida*: advances and prospects. Appl. Microbiol. Biotechnol. 104:7745-7766. <https://doi.org/10.1007/s00253-020-10811-9>.

Xin, X.-F., Kvitko, B., and He, S.Y. (2018). *Pseudomonas syringae*: what it takes to be a pathogen. Nature Rev. Microbiol. 16:316-328. <https://doi.org/10.1038/nrmicro.2018.17>.

Xue, H., Lozano-Durán, R., and Macho, A.P. (2020). Insights into the root invasion by the plant pathogenic bacterium *Ralstonia solanacearum*. Plants. 9:516. <https://doi.org/10.3390/plants9040516>.

Yang, Y., Liu, L., Singh, R.P., Meng, C., Ma, S., Jing, C., Li, Y., and Zhang, C., (2020). Nodule and root zone microbiota of salt-tolerant wild soybean in coastal sand and saline-alkali soil. Front. Microbiol. 11:2178. <https://doi.org/10.3389/fmicb.2020.523142>.

Zboralski, A., and Filion, M. (2020). Genetic factors involved in rhizosphere colonization by phytobeneficial *Pseudomonas* spp. Comp. Struct. Biotech. J. 18:3539-3554. <https://doi.org/10.1016/j.csbj.2020.11.025>.
